# Intricate role of intestinal microbe and metabolite in schizophrenia

**DOI:** 10.1186/s12888-023-05329-z

**Published:** 2023-11-17

**Authors:** Li Shi, Peijun Ju, Xiaojing Meng, Zhongxian Wang, Lihui Yao, Mingming Zheng, Xialong Cheng, Jingwei Li, Tao Yu, Qingrong Xia, Junwei Yan, Cuizhen Zhu, Xulai Zhang

**Affiliations:** 1 Affiliated Psychological Hospital of Anhui Medical University, Hefei, 230022 China; 2 Anhui Clinical Center for mental and psychological diseases, Hefei Fourth People’s Hospital, 316 Mei shan Road, Hefei, Anhui 230000 China; 3 Anhui Mental Health Center, Hefei, 230000 China; 4 Shanghai Mental Health Center, Shanghai key Laboratory of Psychotic Disorders, Shanghai Jiao Tong University School of Medicine, Shanghai, 201108 China; 5 Shenzhen Kangning Hospital, Shenzhen, 518118 China

**Keywords:** Schizophrenia, Gut-brain axis, Microbiome, Metabolomics, Liquid chromatography-mass spectrometry

## Abstract

**Background:**

The brain-gut axis has gained increasing attention due to its contribution to the etiology of various central nervous system disorders. This study aims to elucidate the hypothesis that schizophrenia is associated with disturbances in intestinal microflora and imbalance in intestinal metabolites. By exploring the intricate relationship between the gut and the brain, with the goal of offering fresh perspectives and valuable insights into the potential contribution of intestinal microbial and metabolites dysbiosis to the etiology of schizophrenia.

**Methods:**

In this study, we used a 16S ribosomal RNA (16S rRNA) gene sequence–based approach and an untargeted liquid chromatography-mass spectrometry-based metabolic profiling approach to measure the gut microbiome and microbial metabolites from 44 healthy controls, 41 acute patients, and 39 remission patients, to evaluate whether microbial dysbiosis and microbial metabolite biomarkers were linked with the severity of schizophrenic symptoms.

**Results:**

Here, we identified 20 dominant disturbances in the gut microbial composition of patients compared with healthy controls, with 3 orders, 4 families, 9 genera, and 4 species. Several unique bacterial taxa associated with schizophrenia severity. Compared with healthy controls, 145 unusual microflora metabolites were detected in the acute and remission groups, which were mainly involved in environmental information processing, metabolism, organismal systems, and human diseases in the Kyoto encyclopedia of genes and genomes pathway. The Sankey diagram showed that 4 abnormal intestinal and 4 anomalous intestinal microbial metabolites were associated with psychiatric clinical symptoms.

**Conclusions:**

These findings suggest a possible interactive influence of the gut microbiota and their metabolites on the pathophysiology of schizophrenia.

**Supplementary Information:**

The online version contains supplementary material available at 10.1186/s12888-023-05329-z.

## Introduction

Schizophrenia (SCZ) is a severe neuropsychiatric illness that is prevalent in approximately 1% of the general population worldwide [1], it is now widely acknowledged that its etiology appears to lie in complicated genetic and/or environmental disruption of brain development [2], and the biological mechanisms of the disease are heterogeneous and relatively uncertain [3–5]. In recent years, abundant preclinical and clinical evidence has highlighted the causal role of the gut microbiota in serious mental illnesses, including depression [6], eating disorders [7], Alzheimer’s disease [8], alcohol addiction [9], autism spectrum disorder [10], bipolar disorder [11], and SCZ [12]. These findings have revealed that the gut microbiota comprises a multifarious and dynamic population of microorganisms and is harbored in the gastrointestinal (GI) tract of the host [13]. Additionally, this microbiota could communicate with the brain through complex bidirectional communication systems—the gut-brain axis. Gut microbes could disrupt emotions and the brain functions via neuro–immuno-endocrine mediators, and vice versa, the central nervous system could modulate the peripheral intestinal function via secretion of different biochemicals [14], such as dopamine, serotonin and γ-aminobutyric acid [15]. To date, none of these evidences have been universally accepted, a definitive relationship between the two remains largely elusive, and there is a pressing need to identify precise mechanisms underlying this disorder [16, 17].

Epidemiological studies have found that exposure to increased number of maternal genital/reproductive microbes results in a 10- to 20-fold increased risk of developing SCZ [18]. Animal model research has explored the possible effects of the gut microbiome on neurogenesis, neuronal malfunction, myelination, dendrite formation, and blood brain barrier development [19], and their possible contributions to the pathophysiology of SCZ [20]. The microbiota may modulate the programming of social behavior and cognition, which are known to be disrupted in SCZ [21, 22]. Recently, a total of 9,879,896 genes were identified from 1018 published and 249 newly sequenced samples using next-generation DNA sequencing [23], four phyla of bacteria were found predominantly in the GI, namely *Proteobacteria*, *Actinobacteria, Bacteroides*, and *Firmicutes*, meanwhile, nine genera of bacteria were primary found in GI tract, namely, *Bifidobacterium*, *Escherichia, Prevotella, Eubacterium, Lactobacillus, Clostridium*, *Porphyromonas, Streptococcus* and *Ruminococcus* [24, 25]. In addition, it has been recently found that patients with SCZ have specific differences in the composition and diversity of microbiota compared with healthy controls, for instance, there were fewer numbers of fecal *Bifidobacterium, Escherichia coli*, and *Lactobacillus*, and a significantly higher number of fecal *Clostridium coccoides* in patients with SCZ [26, 27]. Although it is unclear whether these dynamic changes in microbiota are relevant to the severity of clinical symptoms or responsible for host response elicitation, the mechanisms by which these aberrant microbiotas affect the SCZ development chain should be discussed in the future.

More recently, accumulating studies strongly suggest that the imbalance of intestinal microflora could contribute as one of the pivotal factors in the metabolic dysfunctions in SCZ patients [26]. A huge divergence in gut microbiome was also observed between lean and obese individuals, with a 20% higher *Firmicute* count and a 20% lower *Bacteroides* count in lean individuals [28]. In addition, these gut bacteria can generate short-chain fatty acids (SCFAs) metabolites and release different neurotransmitters, for instance, acetylcholine, dopamine, serotonin, and norepinephrine can be produced and released by the *Lactobacillus*, *Bacillus*, and *Enterococcus* species, the *Candida*, *Streptococcus*, *Escherichia*, and *Saccharomyces* species, and the *Escherichia* and *Bacillus* species, respectively [29]. It is conceivable that dysbiosis of intestinal flora metabolites, including neurotransmitters, partly contributes to the pathological causes of SCZ. Considering the abovementioned findings, we hypothesized that anomalous intestinal microflora interaction with metabolic adverse events may exacerbate clinical symptoms in patients with SCZ. This study reveals the associations among the gut microbiota, metabolomics, and clinical phenotypes of SCZ, which sheds new light on enhancing the current understanding of etiological mechanisms of SCZ.

## Materials and methods

### Participants

The Anhui Mental Health Centre (AMHC) approved this study, and the trial clinical registration number was chiCTR1800019343(06/11/2018). Corresponding to the principles of the Declaration of Helsinki, all participants provided signed written informed consent before enrollment. A total of 121 participants were recruited in this study and divided into three groups, among which 44 healthy people were recruited into the control group (healthy controls, n = 44) through the physical examination center of AMHC. According to the Diagnostic and Statistical Manual of Mental Disorders, Fifth Edition (DSM-5), the remaining 80 patients were divided into acute (patients with acute SCZ, n = 41) and remission (patients with SCZ in remission, n = 39) groups. All the patients were hospitalized at AMHC between January 2018 and September 2020 (Fig. S1).

According to the trial standards, all participants were assessed using the Mini-International Neuropsychiatric Interview (MINI) 6.0.0. and patients in the acute and remission groups met the SCZ criteria of the DSM-5. The inclusion criteria for patients in the acute group were as follows: (1) age, 18–60 years; (2) fulfillment of the DSM-5 criteria for the first episode of SCZ without antipsychotic treatment; and (3) Positive and Negative Syndrome Scale (PANSS) total score ≥ 60 points. The inclusion criteria for the patients in the remission group were as follows: (1) age, 18–60 years; (2) fulfillment of the DSM-5 criteria for the SCZ remission stage; (3) only administered second-generation antipsychotic drugs with relatively less metabolic interference, such as risperidone, quetiapine, and aripiprazole, meanwhile, clinical symptoms disappeared after treatment, and the total course of disease was less than 20 years; and (4) PANSS total score < 60. Healthy controls were recruited in the healthy group from the physical examination center of AMHC. The exclusion criteria were as follows: (1) a history of serious organic brain disease, serious physical disease, or other mental disorders; (2) a history of alcohol or other substance use; (3) a history of diabetes, hypertension, dyslipidemia, endocrine diseases, or diseases that may affect metabolism; (4) pregnant or lactating women; (5) a history of digestive tract diseases or intestinal infection in the past 3 months; (6) a history of treatment with antibiotics or corticosteroids, probiotic preparations, or other immune preparations in the past 3 months; and (7) considerable diet change in the past 6 months.

### Clinical assessments

#### Mini-international neuropsychiatric interview (MINI) 6.0.0

Experienced psychiatrists screened participants in the healthy, acute, and remission groups. The initial clinical diagnoses were validated through the Mini-International Neuropsychiatric Interview (MINI). It is a gold standard for the diagnosis of SCZ and has high validity and reliablility. The inter-rater and test–retest reliability were demonstrated with kappa values above 0.80 and 0.90, respectively [30].

#### Positive and negative syndrome scale (PANSS)

The PANSS is extensively used to appraise severe psychopathology in adult patients with SCZ. The versions of the five-factor model have been utilized for the assessment of positive symptoms, negative symptoms, cognitive defects, excited symptoms, and depressive symptoms. It has good reliability and validity, and its Cronbach’s alpha coefficient and intra-class coefficient were 0.928 and 0.878, respectively [31].

#### Fecal sample collection, storage, and processing

Fresh stool samples were collected by trained nurses using a sample collection protocol. Plastic containers with screw-on lids were used to collect stool samples, with Cary-Blair medium is used for transporting stool samples and immediately transferred to the laboratory [32, 33]. They were then stored in a freezer at − 80 °C for future DNA isolation and nucleotide sequencing. After all samples have been collected, they are finally sent to the company on dry ice in a uniform manner for testing.

#### 16 S rRNA gene sequencing analysis

Microbial DNA concentrations in feces were determined through amplification of the 16 S rRNA genes which were extracted using the Qiagen QIAamp Fast DNA stool MINI Kit, according to the manufacturer’s instructions. The V4 hypervariable region of the 16 S rRNA gene was amplified. All selected DNA segments were sequenced in the paired-end mode using an Illumina HiSeq 2500 (Hua da Genomics Technology Service Co., Ltd., Shenzhen, China). Detailed data analysis was performed according to our previously published study [34].

#### Liquid chromatography-mass spectrometry

Each fecal sample (25 mg) was aliquoted into an Eppendorf tube (1.5 mL) with 10 µL of internal standard and 800 µL of a mixture extract solution, including 360 µL of methanol, 360 µL of acetonitrile, and 160 µL of water. The mixture was sonicated for 10 min at 4 °C, and placed in a refrigerator at -20 ℃ for 1 h after homogenization, and then centrifuged at 25,000 g for 15 min. Next, 600 µL of supernatant fluid was used for liquid chromatography-mass spectrometry (LC-MS) analysis. Every eighth quality control sample was injected to assess the repeatability of the experiments. Waters 2D UPLC (Waters, USA) and Q Exactive high-resolution mass spectrometer (Thermo Fisher Scientific, USA) were used to separate and detect the positive and negative ion coverage of the metabolites. The internal standards contain mainly D3-L-Methionine (100 ppm, TRC, Canada), 13C9-Phenylalanine (100ppm, CIL, USA), D6-L-2-Aminobutyric Acid (100ppm, TRC, Canada), D4-L-Alanine (100ppm, TRC, Canada), Threonine (100ppm, CIL, USA), D3-L-Aspartic Acid (100ppm, TRC, Canada), 13C6-L-Arginine (100ppm, CIL, USA). Relevant solvents used in the experiment, such as methanol (A454-4), acetonitrile (A996-4) were LCMS grade (Thermo Fisher Scientific, USA); ammonia formate (17843-250G, Honeywell Fluka, USA), formic acid (50144-50 ml, DIMKA, USA), and water were obtained from a pure water instrument supplied. The LC-MS experimental conditions were divided into chromatographic and mass spectrometric conditions. For the chromatographic conditions, the column used in this experiment was a BEH C18 column (1.7 μm 2.1*100 mm, Waters, USA). The mobile phases in positive ion mode were aqueous solution containing 0.1% formic acid (liquid A) and 100% methanol containing 0.1% formic acid (liquid B), while the mobile phases in negative ion mode were aqueous solution containing 10 mM ammonia formate (liquid A) and 95% methanol containing 10 mM ammonia formate (liquid B). The elution was carried out using the following gradients: 0 ~ 1 min, 2% B solution; 1 ~ 9 min, 2%~98% B solution; 9 ~ 12 min, 98% B solution; 12 ~ 12.1 min, 98% B solution to 2% B solution; 12.1 ~ 15 min, 2% B solution. The flow rate was 0.35 mL/min, the column temperature was 45 °C, and the injection volume was 5 µL. In additional, in the mass spectrometry conditions, the Q Exactive mass spectrometer (Thermo Fisher Scientific, USA) was used for primary and secondary mass spectrometry data acquisition. The mass to nucleus ratio of the mass spectrometry scans ranged from 70 to 1050, with a primary resolution of 70,000, an AGC of 3e6 and a maximum injection time (IT, injection time) of 100 ms. The secondary information was collected by selecting Top3 for fragmentation according to the parent ion intensity, with a secondary resolution of 17,500, an AGC of 1e5, a maximum injection time (IT. injection time) was set to 50 ms and the stepped nce was set to 20, 40 and 60 eV. The ESI parameters were set to 40 for the Sheath gas flow rate, 10 for the Aux gas flow rate, and 10 for the Spray voltage (|KV|). voltage(|KV|)) of 3.80 for positive ion mode and 3.20 for negative ion mode, ion transfer tube temperature (Capillary temp) of 320 °C and Aux gas heatertemp of 350 °C. The LC-MS data were processed using Compound Discoverer 3.1 (Thermo Fisher Scientific, USA) software (Hua da Genomics Technology Service Co., Ltd., Shenzhen, China). We obtained fecal metabolome data from 9 healthy volunteers, 10 SCZ patients from the acute group, and 10 SCZ patients from the remission group. The R software package (meta X) was used to visualize the data pre-processing, metabolite differences, metabolite taxonomic annotation, and functional annotation from metabolomics among the three groups [35]. Pathway enrichment analysis was conducted using the MetaboAnalyst [36], Kyoto Encyclopedia of Genes and Genomes (KEGG) [37], and Human Metabolome Databases (HMDB) [38].

### Statistical analysis

#### Demographic analysis

SPSS version 25.0, statistical package for Windows was used for demographic and clinical characteristics of the data among the three groups. Continuous variables such as age, body mass index, and symptom scores were analyzed using an independent samples t-test, and discontinuous variables were compared using the χ 2 test. A one-way analysis of variance (ANOVA) followed by LSD multiple comparison tests was used to analyze differences among the three groups [39, 40].

#### Microbiome analyses

To explore the core diversity metrics of the alpha-diversity index of Ace, Chao, Coverage, Shannon, Simpson, and Sobs, we used GraphPad Prism version 8 to calculate a range of estimators. The beta diversity index was calculated using weighted UniFrac metric analysis and Principal Component Analysis (PCA). The Kruskal–Wallis test was used to analyze the divergence concentration of fecal microbial and fecal metabolites among the three groups and complemented by the Bonferroni correction for pairwise comparisons. Heat maps and network plots of the bias correlation coefficients of the two variables were filtered using the V-search (version v3.5.0) and R (version v3.6.1) software. Kyoto Encyclopedia of Genes and Genomes (KEGG) predicted functional pathways based on the MetaCyc Metabolic Pathway Database. A Sankey plot graph was constructed to facilitate understanding of the complex flow situations of anomalous fecal microbes, fecal metabolites, and clinical symptoms. Statistical significance was set at *P* < 0.05.

## Results

### Clinical characteristics of study participants

A total of 121 participants were included in the final analyses and were divided into three groups: healthy (n = 44), acute (n = 41), and remission (n = 39). First, the baseline demographic and clinical characteristics of this study are presented in Table 1. The results showed no statistical differences among the three groups in terms of baseline demographic and clinical characteristics, namely, age, sex, body mass index, year of education, duration of illness, and equivalent dose of psychotropic drugs (*P* > 0.05). The acute and remission groups underwent PANSS assessments conducted by experienced psychiatrists. Unsurprisingly, the total PANSS (73.07 ± 11.30 vs. 45.41 ± 6.82, *P* = 0.002) and subscale scores for positive, negative, cognitive defect, excited, and depressive symptoms (13.63 ± 6.05 vs. 9.56 ± 2.34, *P* < 0.001; 17.90 ± 7.85 vs. 13.54 ± 4.23, *P* = 0.014; 10.10 ± 3.53 vs. 6.69 ± 1.84, *P* = 0.009; 10.15 ± 3.90 vs. 5.69 ± 1.10, *P* < 0.001; 4.07 ± 2.13 vs. 3.10 ± 0.68, *P* = 0.001) were worse in the acute group than in the remission group.

**Table 1:** Comparison of demographic and clinical data between three groups

Factors	Healthy group(n = 44)	Acute group (n = 41)	Remission group (n = 39)	F/t/χ2	*P*
Age(years)	42.61 ± 11.81	38.59 ± 11.25	39.74 ± 11.17	1.406	0.249
Sex(Males/Females)				2.363	0.307
Males(proportion%)	23(52.3)	17(41.5)	14(35.9)		
Females(proportion%)	21(47.7)	24(58.5)	25(64.1)		
BMI (kg/m2)	22.68 ± 3.06	23.66 ± 2.78	23.85 ± 3.68	1.647	0.197
Years of education(years)	10.44 ± 4.38	10.51 ± 4.38	9.72 ± 3.90	0.892	0.412
Course of disease(years)		10.60 ± 7.49	13.01 ± 8.74	-1.322	0.090
Drugs					
CPZ equivalent doses(mg)		171.44 ± 121.68	227.99 ± 134.99	-1.970	0.181
PANSS					
Positive symptom		13.63 ± 6.05	9.56 ± 2.34	3.934	0.000
Negative symptom		17.90 ± 7.85	13.54 ± 4.23	3.071	0.014
Cognitive defect symptom		10.10 ± 3.53	6.69 ± 1.84	5.366	0.009
Excited symptom		10.15 ± 3.90	5.69 ± 1.10	6.091	0.000
Depressive symptom		4.07 ± 2.13	3.10 ± 0.68	2.721	0.001
PANSS-Total		73.07 ± 11.30	45.41 ± 6.82	7.279	0.002

Note: BMI: Body mass index; CPZ: chlorpromazine; PANSS: Positive and Negative Syndrome Scale

### Significantly different intestinal flora and fecal metabolites in the acute, remission and healthy control groups

**Fig. 1:**
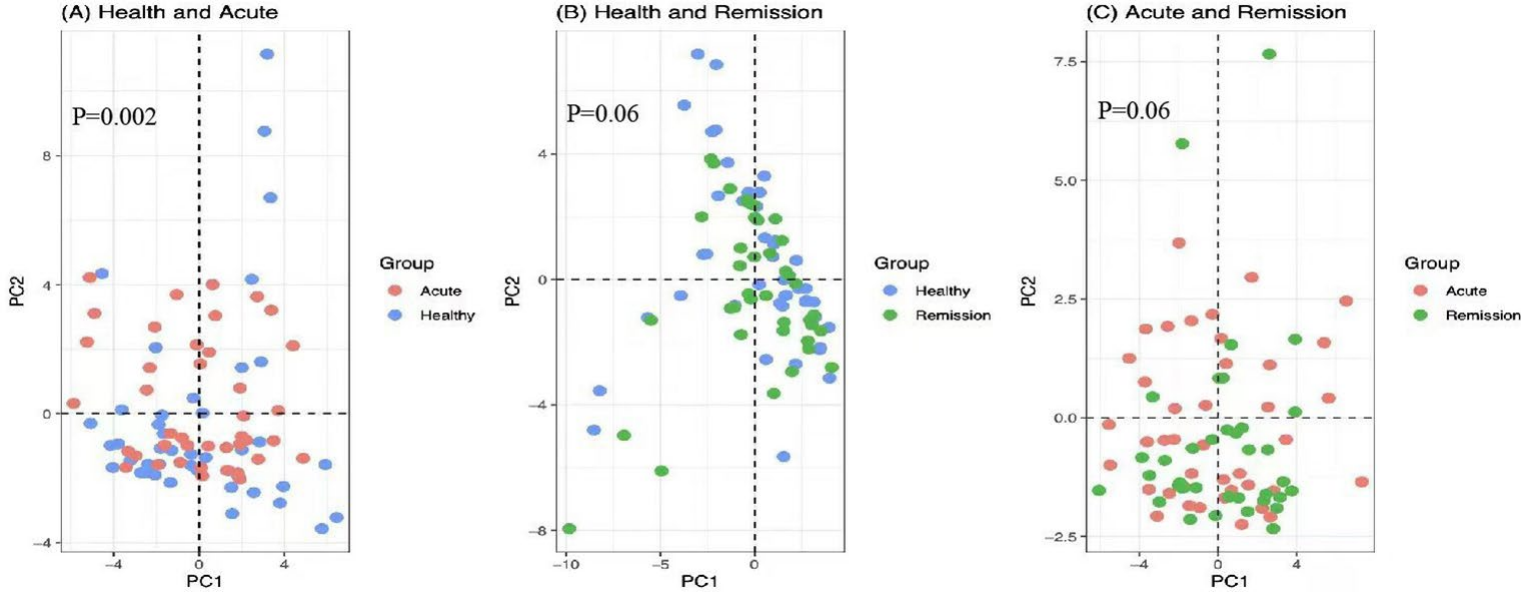
Principal component analysis. PCA plots illustrating the beta-diversity distance matrix of Bray-Curtis distances comparing the distribution characteristics of the samples in different groups. The blue, red and green dots represent the healthy, acute and remission groups, respectively. (**A**) Acute group and Healthy group; (**B**) Remission group and Healthy group; (**C**) Acute group and Remission group

**Fig. 2:**
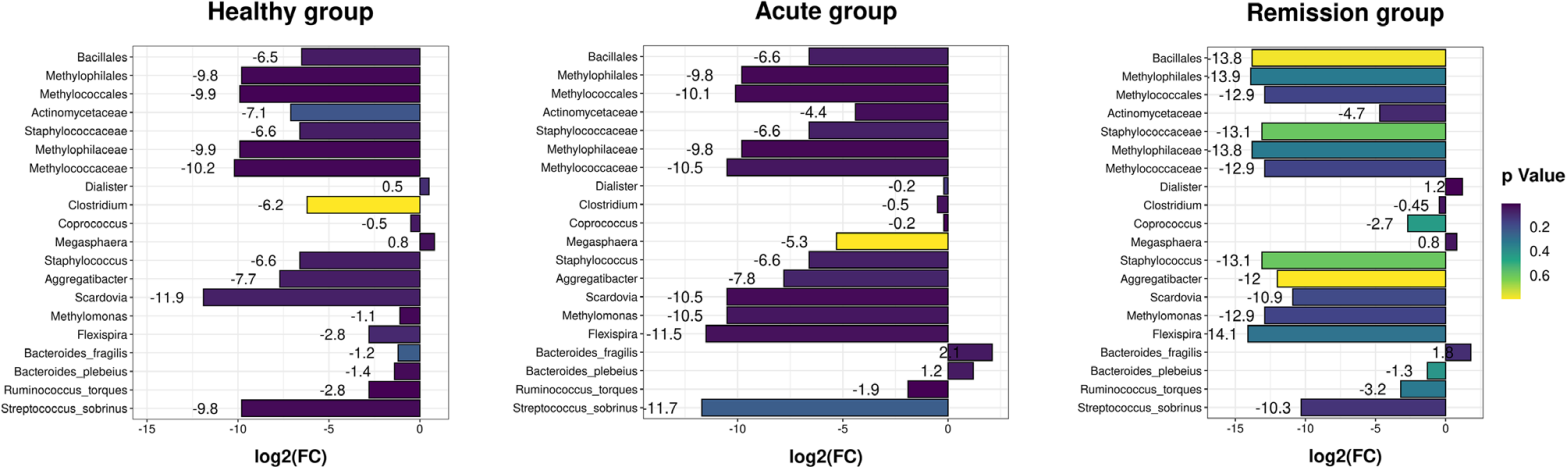
20 abnormal bacteria in their difference abundance fold change values(log2FC) as detected among three group. (**A**) Healthy group; (**B**) Acute group; (**C**) Remission group. The expressing higher enriched levels of bacteria abundance are shown in the dark blue, whereas those expressing lower enriched levels of bacteria abundance are shown in the green and yellow

To estimate the discrepant richness and diversity of the microbial community structure, we calculated the alpha and beta metrics. The alpha diversity indices of Ace (*P* = 0.478), Chao (*P* = 0.504), Coverage (*P* = 0.661), Shannon (*P* = 0.342), Simpson (*P* = 0.684), and Sobs (*P* = 0.929) did not show any divergence among the three groups (Fig. S2). PCA analysis revealed that the beta diversity composition of gut flora was diverse between the healthy and acute groups (*P* = 0.002), while the results did not show differences between the healthy and remission groups (*P*=0.06) or the acute and remission groups (*P* = 0.06) (Fig. 1). Moreover, to detect the specific taxonomic alterations of gut microbiota, we analyzed the microflora relative abundance changes of phyla, class, order, family, genus, and species among the three groups; ultimately, we identified 20 dominant distinguishable intestinal microflora, which included *Bacillaes*, *Methylophilales*, and *Methylococcales* of three orders, *Actinomycetaceae, Staphylococcaceae, Methylophilaceae*, and *Methylococcaceae of four families, Dialister, Clostridium, Coprococcus, Megasphaera, Staphylococcus, Aggregatibacter, Scardovia, Methylomonas, and Flexispira* of nine genus, and *Bacteroides_fragilis, Bacteroides_plebeius, Ruminococcus_torques, and Streptococcus_sobrinus* of four species (Fig. 2).

Compared with the healthy groups, at the family level, the abundance of *Actinomycetaceae* was higher in the acute group (*P* = 0.011). At the genus level, the abundance of *Aggregatibacter* (*P* = 0.038, *P* = 0.031), *Methylomonas* (*P* = 0.016, *P* = 0.005), and *Flexispira* (*P* = 0.016, *P* = 0.046) was lower in both the acute and remission groups, and the abundance of *Dialister* (*P* = 0.005) and *Megasphaera* (*P* = 0.012) in the acute groups was lower compared with the remission groups. At the species level, the abundance of *Bacteroides_ fragilis* (*P* = 0.020) and *Ruminococcus_torques* (*P* = 0.005) was higher in the acute group. We also found that the abundance of *Bacteroides_fragilis* increased in the acute group compared with the remission group (*P* = 0.044) (Fig. 2).

**Fig. 3:**
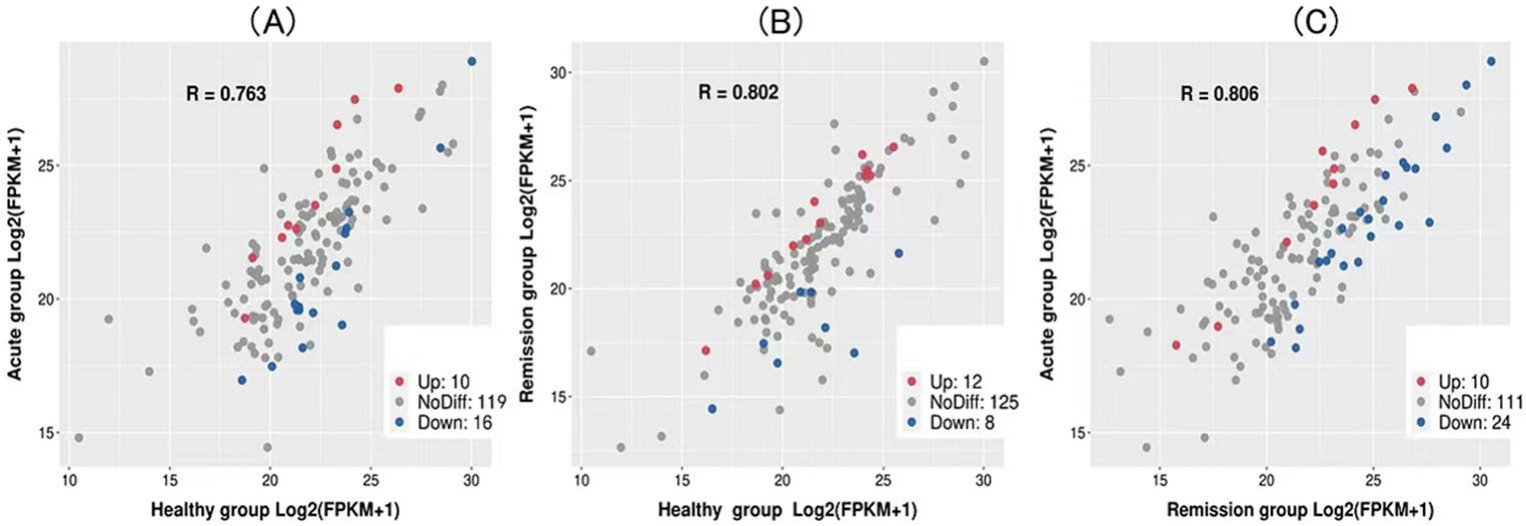
Scatter diagrams show the relationship between changes in the overall metabolites. Red dots and blue dots represent upregulated and downregulated metabolites respectively, grey dots represent no-different significantly metabolites. (**A**) Acute group VS Healthy group; (**B**) Remission group VS Healthy group; (**C**) Acute group VS Remission group

By comparing the levels of metabolites, we identified 10,507 and 2853 compounds with identification information generated in the faces of the three groups of participants. Moreover, we detected 145 unusual gut microflora metabolites at substantially different modes in the acute, remission, and healthy groups, and found that the levels of 10 gut microflora metabolites increased and 16 decreased in the healthy groups compared with the acute groups (Fig. 3A**)**, while 12 gut microflora metabolites were elevated and 8 decreased in remission groups compared with the healthy group (Fig. 3B). Interestingly, we found that 10 metabolites were up-regulated and 24 were down-regulated in the acute group compared to the remission group (Fig. 3C). For example, compared with the healthy group’s lipid metabolites and oxidation metabolites, such as Docosapentaenoic acid(*P* = 0.005), Testosterone(*P* = 0.008), Desoxycortone(*P* = 0.022), 8z,11z,14z-eicosatrienoic acid(*P* = 0.026) and Sphingosylphosphorylcholine (*P* = 0.029) exhibited an increase in the acute groups (Table S1).

### Correlation between abnormal intestinal microorganisms with clinical symptoms

**Fig. 4:**
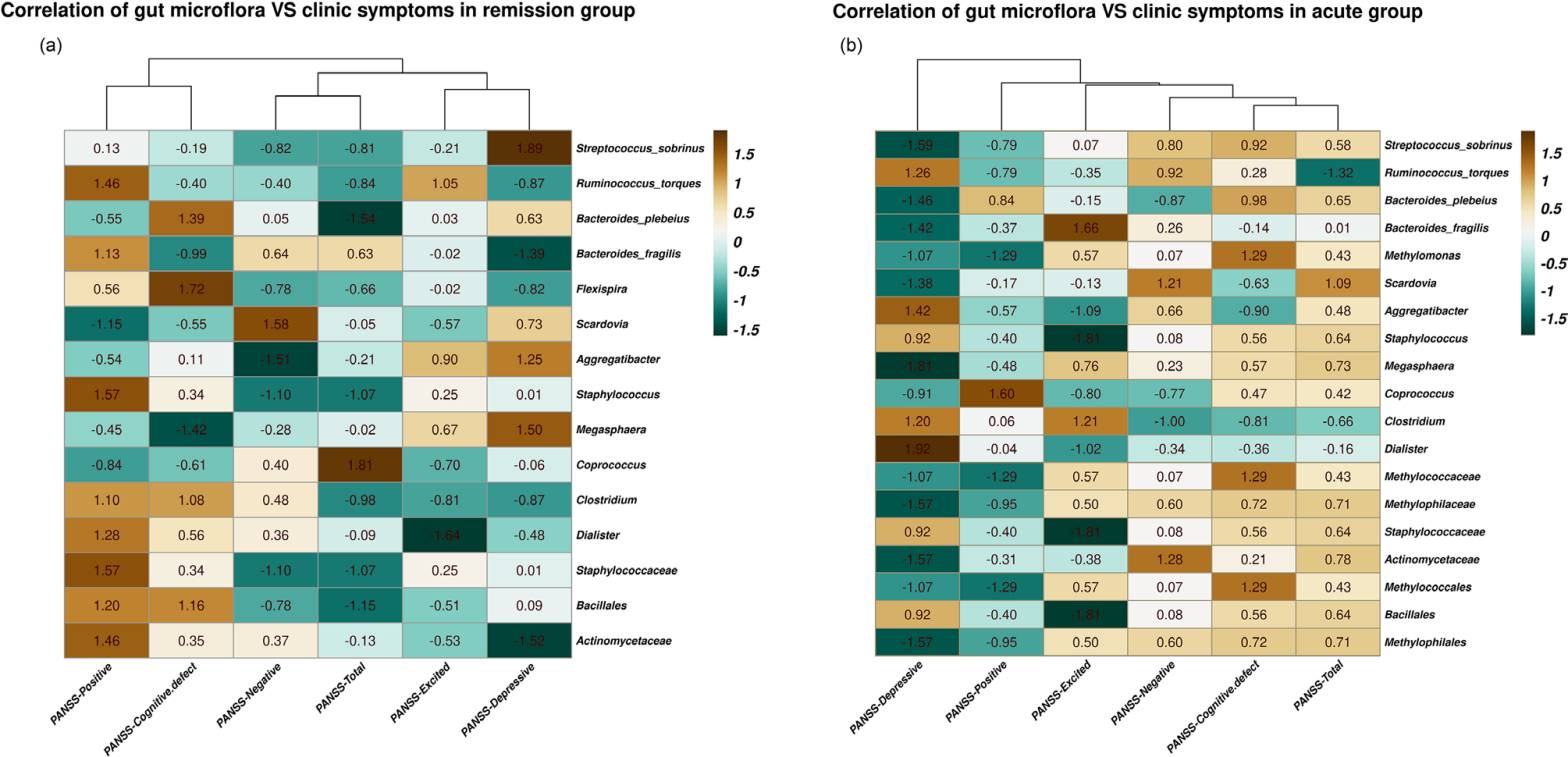
Correlations heatmap of gut microbes and clinical symptoms. (**A**) in remission group; (**B**) in acute group

To investigate the relationship of these dysregulated intestinal microorganisms and their metabolites with clinical symptoms, we performed a Pearson correlation analysis to determine these unbalanced markers and the five factors of PANSS in the acute and remission groups, respectively (Fig. 4). In the remission group, at the family level, *Actinomycetaceae* was found to have a negative correlation with depressive symptoms (*r*=-1.52, *P* = 0.034); at the genus level, *Clostridium* showed a negative correlation with excited symptoms, depressive symptoms, and PANSS-Total (*r*=-0.81, *P* = 0.044; *r*=-0.87, *P* = 0.039; *r*=-0.98, *P* = 0.032) (Fig. 4A). In the acute group, at the order and family level, *Methylophilales*(*r* = 0.60,*P* = 0.001;*r* = 0.72,*P*<0.001;*r* = 0.50, *P* = 0.001;*r* = 0.71, *P*<0.001) and *Methylophilaceae*(*r* = 0.60, *P* = 0.001;*r* = 0.72, *P*<0.001;*r* = 0.50, *P* = 0.001;*r* = 0.71, *P*<0.001) both were positively correlated with negative symptoms, cognitive defect symptoms, excited symptoms, and PANSS-Total. At the genus level, *Dialister* was positively correlated with depressive symptom (*r* = 1.92, *P* = 0.004), *Clostridium* had a negative relationship with negative symptoms, cognitive defect symptoms, and PANSS-Total (*r*=-1.00, *P* = 0.015;*r*=-0.81, *P* = 0.030;*r*=-0.66, *P* = 0.048), and *Megasphaera* was positively related to negative symptoms, cognitive defect symptoms, excited symptoms, and PANSS-Total (*r* = 0.23, *P* = 0.012;*r* = 0.57, *P* = 0.002;r = 0.76, *P* = 0.001;*r* = 0.73, *P* = 0.001). Additionally, at the species level, *Bacteroides_plebeius* showed a positive correlation with positive symptoms and cognitive defect symptoms, and PANSS-Total (*r* = 0.84, *P* = 0.003;*r* = 0.98, *P* = 0.002;*r* = 0.65, *P* = 0.005) and *Ruminococcus_torques* were negatively correlated with positive symptoms and PANSS-Total (*r*=-0.79, *P* = 0.037;*r*=-1.32, *P* = 0.015) and *Streptococcus_sobrinus* showed a positive association with negative symptoms, cognitive defect symptoms, and PANSS-Total (*r* = 0.80, *P* = 0.010;*r* = 0.92, *P* = 0.006;*r* = 0.58, *P* = 0.025) (Fig. 4B). As well as, we observed abnormal gut microbiota were related to demography data, such as education level and course of disease etc. The results are presented in Table S2 and Table S3 of supplementary materials.

### Correlation between anomalous intestinal microbial metabolites with clinical symptoms

**Fig. 5:**
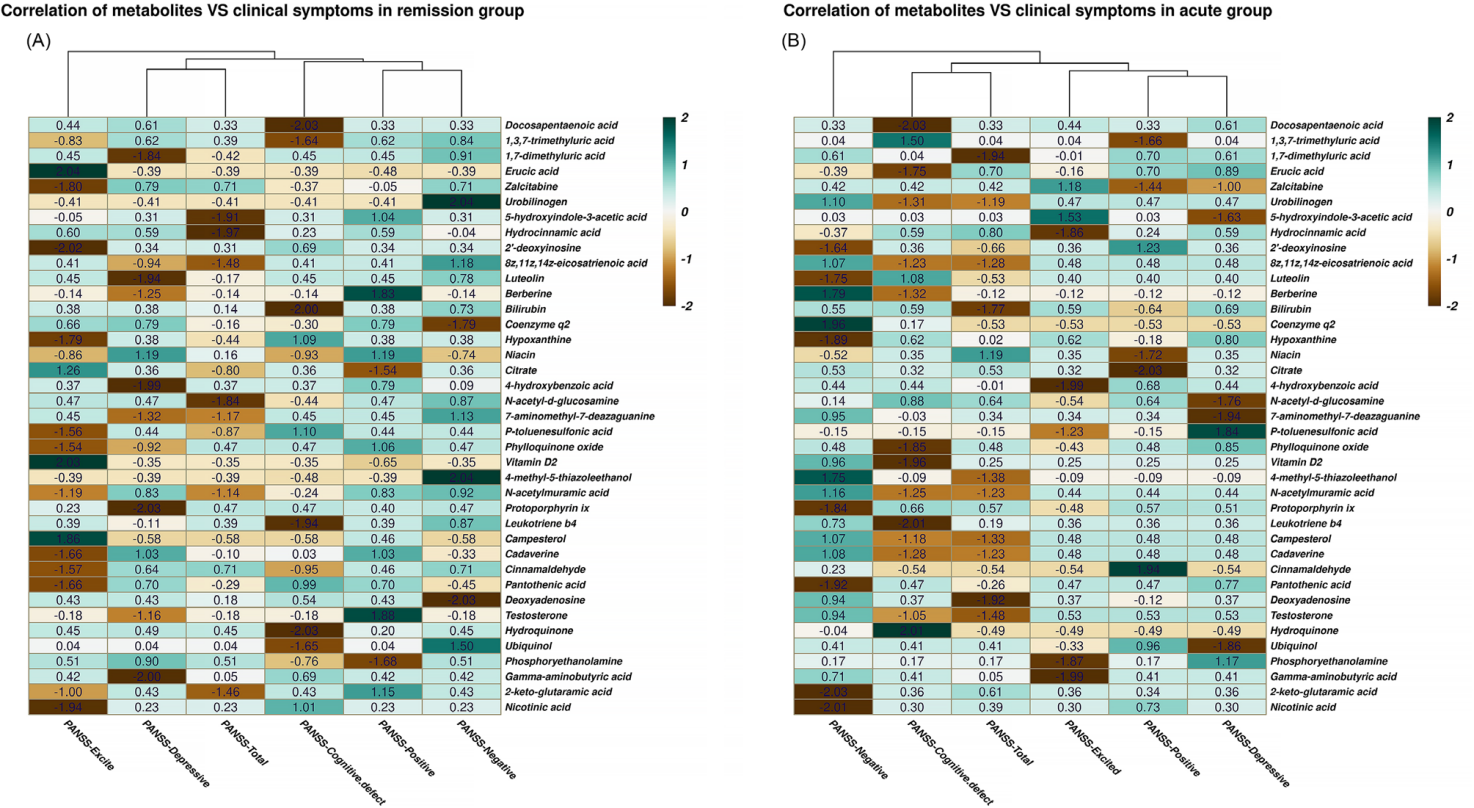
Correlations heatmap of faces metabolites and clinical symptoms. (**A**) in remission group; (**B**) in acute group

The same analyses were used to determine the correlation between unusual intestinal microbial metabolites and psychiatric symptoms (Fig. 5). In the remission group, we observed that 11 anomalous intestinal microbial metabolites were associated with psychiatric symptoms. 1,3,7-trimethyluric acid showed a positive correlation with negative symptoms (*r* = 0.84, *P* = 0.002). Zalcitabine was positively correlated with positive symptoms, and depressive symptoms (*r*=-0.05, *P* = 0.018;*r* = 0.79, *P*<0.001). 2’-deoxyinosine was positively correlated with cognitive defect symptoms and PANSS-Total (*r* = 0.69, *P* = 0.030; *r* = 0.31, *P* = 0.048). Hypoxanthine was positively correlated with cognitive defect symptoms (*r* = 1.09, *P* = 0.036). P-toluenesulfonic acid had a positive correlation with cognitive defect symptoms, and PANSS-Total (*r* = 1.10, *P* = 0.001; *r*=-0.87, *P* = 0.037), Protoporphyrin ix was positive correlated with positive symptoms (*r* = 0.40, *P* = 0.047). Cinnamaldehyde showed a positive relationship with positive symptoms (*r* = 0.46, *P* = 0.004). (Fig. 5A).

In the acute group, we observed that 9 anomalous intestinal microbial metabolites were associated with psychiatric symptoms, such as vitamin D2 was negatively correlated with positive symptoms (*r* = 0.25, *P* = 0.042), and 7-aminomethyl-7-deazaguanine was negatively associated with positive symptoms (*r* = 0.34, *P* = 0.030). Moreover, we also found that four metabolites showed a positive correlation with psychiatric symptoms, namely, Pantothenic acid had a positive correlation with depression symptoms (*r* = 0.77, *P* = 0.025), 4-methyl-5-thiazoleethanol had a positive correlation with the negative symptoms (*r*=-1.75, *P* = 0.044), Luteolin with cognitive defect symptoms (*r* = 1.08, *P* = 0.009), and Niacin had a positive correlation with the PANSS-Total (*r* = 1.19, *P* = 0.025) (Fig. 5B). Meanwhile, we found some unusual metabolites were linked to demography data such as gender and course of disease etc. The results are presented in Table S4 and Table 5 of supplementary materials.

### Predictive functional analysis about metabolic pathways of anomalous intestinal microbial metabolites

**Fig. 6:**
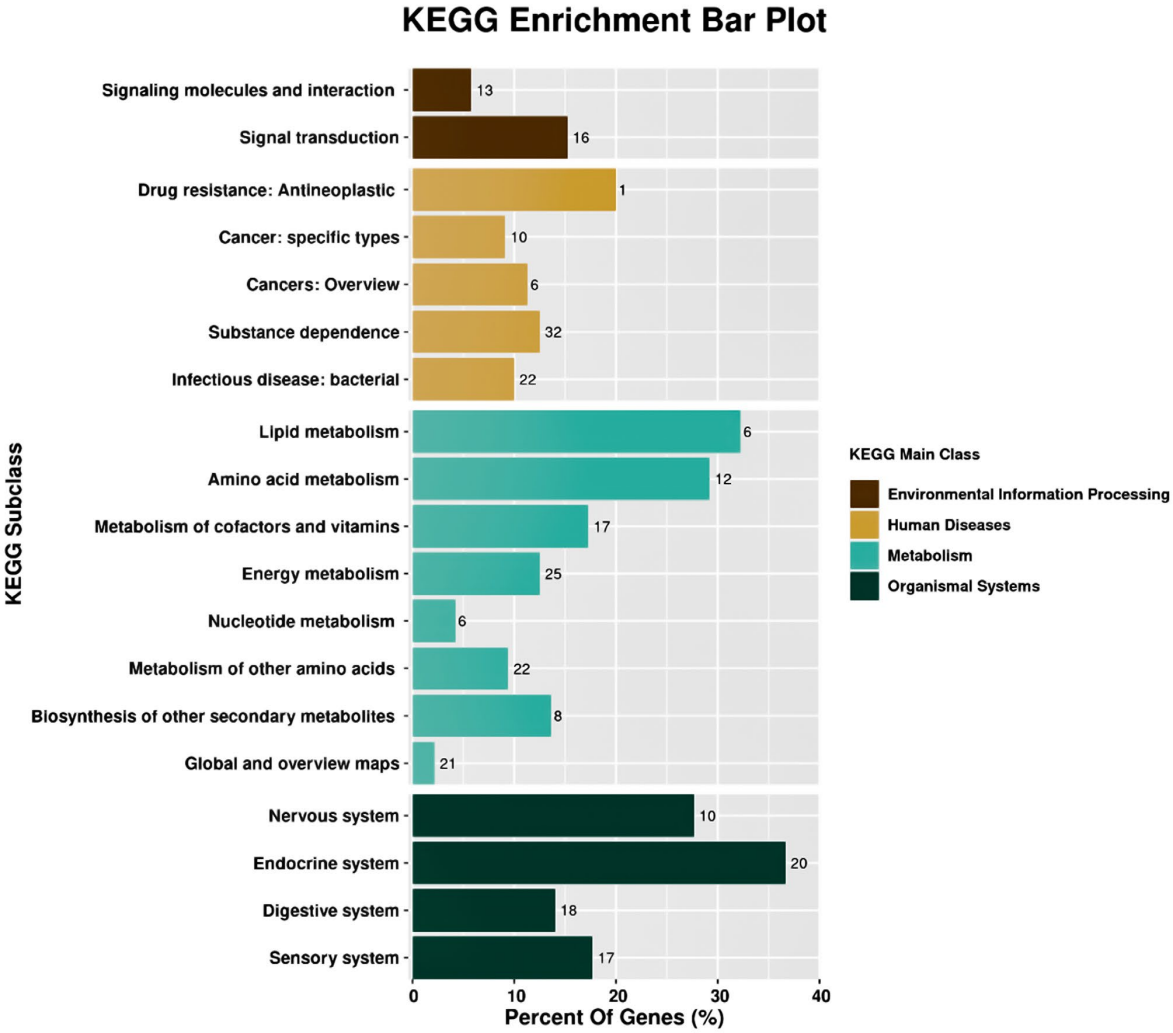
KEGG enriched pathways of changed fecal metabolites. The percent of genes is the ratio of the proportion of changed metabolites in the pathway and the proportion of all metabolites in the pathway. The larger the value is, the greater the enrichment degree is

We further investigated gene functions and linked genomic information with higher-order functional information of these anomalous intestinal microbial metabolites. We analyzed these abnormal intestinal microbial metabolites using the Kyoto Encyclopedia of Genes and Genomes (KEGG) database to confirm the cellular processes and standardize gene annotations [37], which was used to analyze the related metabolic pathways. For the molecular function of KEGG subclass, the hub genes were found to be mainly related to signaling molecules and interaction, signal transduction, antineoplastic drug resistance, cancer of specific types, lipid metabolism, metabolism of cofactors and vitamins, amino acid metabolism, sensory system, nervous system, digestive system, endocrine system, etc. (Fig. 6). The results of KEGG pathway analysis highlighted that the hub genes were mainly concentrated on environmental information processing, metabolism, organismal systems, and human diseases (Fig. 6).

### Multi-omic network analysis reveals the interaction among abnormal intestinal microbes, metabolites and clinical symptoms

**Fig. 7:**
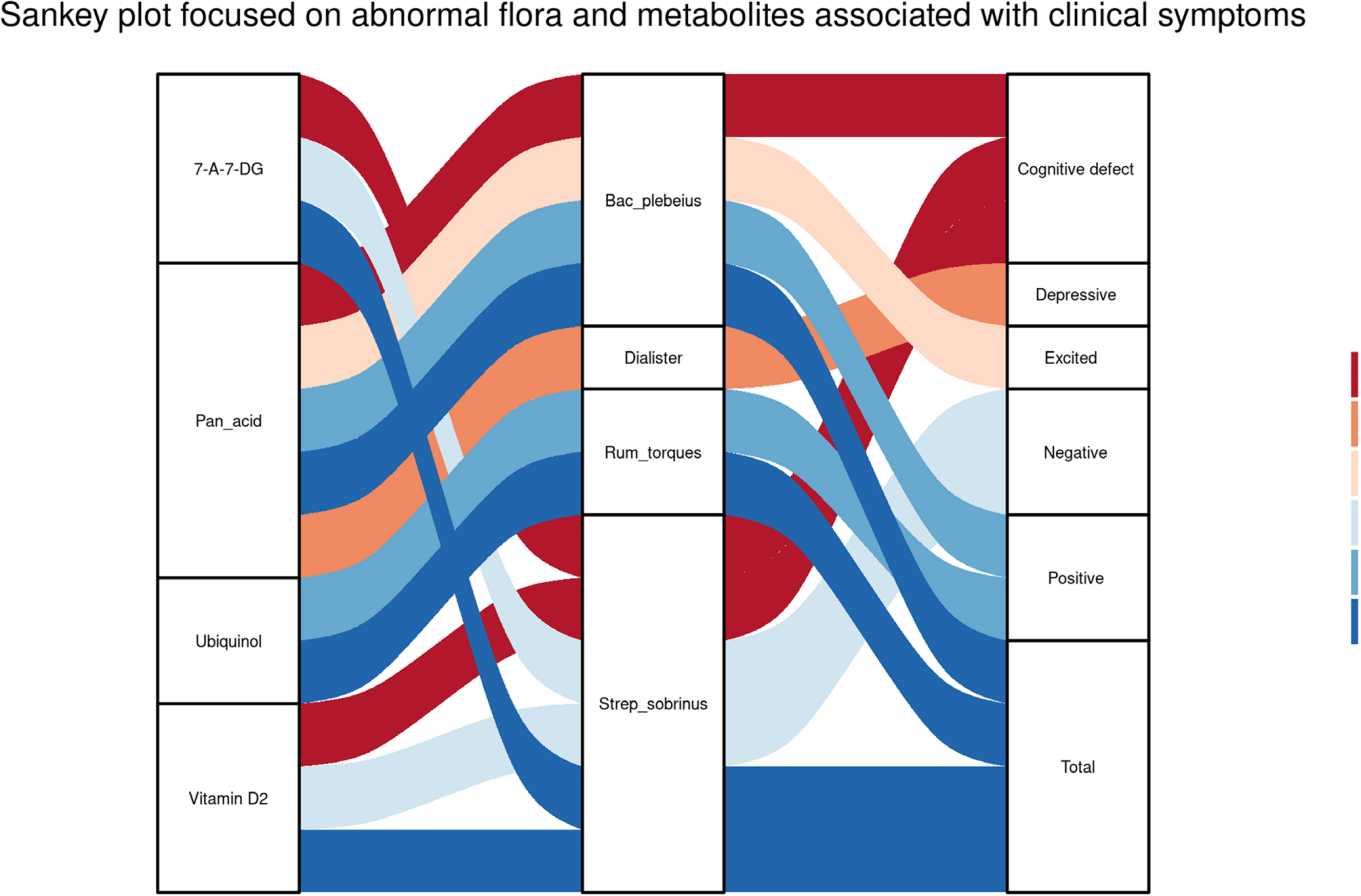
Sankey plot focused on anomalous floras and metabolites with clinical symptoms. The leftmost side represents metabolites, the middle represents intestinal flora, and the rightmost side represents psychiatric symptoms. 7-A-7-DG: 7-aminomethyl-7-deazaguanine, pan_acid: Pantothenic acid, Bac_plebeius: Bacteroides_plebeius,Rum_torques:Ruminococcus_torques,Strep_sobrinus:Streptococcus_sobrinus.

Based on the above-mentioned results, we used the Sankey diagram to visualize the potential interactive effect among abnormal intestinal microorganisms, microbial metabolites and psychiatric symptoms. The Sankey diagram constructed the co-expression network among the screened four anomalous intestinal microbial metabolites, four abnormal intestinal microorganisms and psychiatric clinical symptoms with Pearson correlation analysis (|R| > 0.4 and *P* < 0.05). For example, the Sankey plot showed that 7-aminomethyl-7-deazaguanine and vitamin D2 were related to *Streptococcus_sobrinus* (*r* = 0.77, *P* = 0.009; *r* = 0.648, *P* = 0.043); simultaneously, *Streptococcus*_*sobrinus* was a crucial effector associated with cognitive defect symptoms (*r* = 0.42, *P* = 0.006) (Fig. 7).

## Discussion

The interaction between the gut microbiota and microbial metabolites can influence brain function and behavior through the gut-brain axis, and thus may predispose the development of SCZ. Here, we found profound alterations in the gut microbiota at the order, family, genus, and species levels in patients with SCZ relative to healthy subjects. We identified unique discrepant bacterial taxa that were strongly associated with the different disease states of SCZ severity. Moreover, a specific diversity in intestinal microbial metabolite panel was found in patients with SCZ relative to healthy subjects, and these abnormal intestinal microbial metabolites were closely related to SCZ clinical symptomatology. The Sankey diagram constructed the co-expression network to enable us to identify networks of four anomalous intestinal microbial metabolites and four abnormal intestinal microorganisms associated with psychiatric clinical symptoms. These findings suggest a possible interactive influence of the gut microbiota and their metabolites on the pathophysiology of SCZ.

The GI tract is a complex ecological system containing a large number of resident microorganisms [41]. Recent evidence suggests that alterations in gut microbial composition are implicated in the pathophysiology of psychiatric clinical syndromes [42, 43]. Feng Zhu found that many facultative anaerobes such as *Lactobacillus fermentum, Enterococcus faecium, Alkaliphilus oremlandii*, and *Cronobacter sakazakii/turicensis*, harbored in SCZ patients, are rare in a healthy gut, and transplantation of a SCZ-enriched *Streptococcus vestibularis*, appears to induce social behavior deficits and neurotransmitter level alterations in recipient mice [26]. Further clinical research indicated that increased *Lactobacillus* bacteria were significantly correlated with the severity of different symptom domains in first-episode SCZ patients[44]. Schwartz and colleagues also observed a relatively increased abundance of the families, *Lactobacillaceae, Halothiobacillaceae, Brucellaceae*, and *Micrococcineae*, and decreased abundance of *Veillonellaceae* in first-episode SCZ patients compared to control subjects; in particular, *Lactobacillaceae* were overrepresented and most strongly increased in SCZ patients [44]. A promising candidate curative treatment that involves taking probiotic supplements may help normalizing the *C. albicans* antibody and *C. albicans* levels in many gut discomfort male individuals and ameliorate psychiatric symptoms through correcting *C. albicans* abundance [45]. To date, studies on the intestinal microbe of SCZ are mostly focused on first-episode patients, and are rare in patients with different phenotypes of SCZ. In this study, we investigated the unique gut microbial alteration of different clinical phenotypes of SCZ. Coinciding with earlier experiments, we found no discrepancy in alpha diversity among the three groups, while the beta diversity differed between the healthy and acute groups, but not the remission group [44]. 20 dominant diversity intestinal microflora were identified, including three orders, four families, nine genera, and four species among the three groups; in particular, the abundance of *Actinomycetaceae, Aggregatibacter, Bacteroides_ fragilis, and Ruminococcus_torques* was the highest in the acute group, and the abundance of *Dialister* and *Megasphaera* was the lowest in the acute group. Moreover, these anomalous intestinal microflora had a significant correlation with the severity of different symptom domains in patients with SCZ with different clinical phenotypes.

Dysregulation of neural neurotransmitters has been widely recognized as a hallmark of SCZ [19]. Previous research has demonstrated that gut microbes and their metabolites may penetrate the systemic circulation and access the brain, and these differential metabolites exhibit consistent alterations in the brain region[46]. These unusual metabolites are mainly involved in glycerophospholipid and fatty acyl metabolism, and the anomalous glycerophospholipid and fatty acyl metabolism are implicated in the onset of SCZ-relevant symptoms, which may provide a new understanding of the etiology of SCZ [46]. In the present preclinical and pioneering studies, almost ten metabolomics involved patients with SCZ were identified, namely N-acetyl aspartate, lactate, tryptophan, kynurenine, glutamate, creatine, linoleic acid, D-serine, glutathione, and 3-hydroxybutyrate [47]. We showed that there were 145 dysbiosis metabolomics among the three groups, among which 11 anomalous intestinal microbial metabolites of the remission group and 9 anomalous intestinal microbial metabolites of the acute group were correlated with psychotic symptoms. Focusing on unusual intestinal microbes and their metabolomics, we observed that these anomalous molecules were mainly involved in environmental information processing, metabolism, organismal systems, and human diseases. Interestingly, the interaction between four anomalous intestinal microbial metabolites and four abnormal intestinal microorganisms highlighted their association with psychiatric clinical symptoms. This finding highlights the key to breaking the deadlock of the SCZ pathological mechanism, which may involve the intestinal microbiota and intestinal microbial metabolites.

This study has several strengths. First, the primary emphasis of this research lies in exploring the interactive relationship among the gut microbiota, metabolites associated with psychotic symptoms during both acute and remission stages. It effectively captures the dynamic variations within the gut environment under distinct pathological conditions. Secondly, we detected a correlation between certain gut bacteria, such as *Streptococcus-Sobrinus*, and metabolic byproducts like 7-aminomethyl-7deazaguanine and vitamin D2. Furthermore, we observed a strong connection between these bacteria and the cognitive function of patients. Pathway analysis highlighted that the hub genes were mainly concentrated on environmental information processing, metabolism, organismal systems, and human diseases. These significant findings open up new avenues for treatment and intervention of SCZ. The results of the present study should be interpreted without overlooking its limitations. First, the main limitation of this study was its small sample size; therefore, the findings should be considered preliminary and need to be reproduced in larger independent samples. Second, it is not possible to conclude causality using the case-control study design, and the interactive effects of abnormal intestinal flora and their metabolites on reported findings could be a potential influence alone. Finally, microbiota composition could be affected by diet; therefore, more detailed dietary data would have been needed to conclusively exclude bias.

## Conclusions

In summary, our study revealed significant beta differences in the microbial composition, panning various taxonomic levels such as family, order, genera, and species, as well as microbial metabolites, among acute patients, remission patients and healthy controls. These differences were found to be link with severity of symptoms. Pathway analysis highlighted the prominence of hub genes related to environment information processing, metabolism, organismal systems, and human diseases. Additionally, we observed interactive correlations between four abnormal intestinal microbial metabolites and four anomalous intestinal microorganisms, further underscoring their association with psychiatric clinical symptoms. Replication studies and investigation into the underling mechanisms of SCZ are warranted to gain deeper insights into the potential benefits of microbiota and the modulation of their metabolites on psychiatric symptoms.

### Electronic supplementary material

Below is the link to the electronic supplementary material.

Supplementary Material 1: Flow chart of the experiment and comparison of fecal metabolite content and alpha diversity of the gut microbiota between the three groups.

Supplementary Material 2： Correlation between altered gut microbiota and fecal metabolites and demographic data in SCZ patients in the acute and remission groups, respectively.

## Data Availability

The data that support the findings of this study are available from Hefei Fourth People’ Hospital but restrictions apply to the availability of those data, which were used under license for the current study, and so are not publicly available. Data are however available from the authors upon reasonable request and with permission of Hefei Fourth People’ Hospital. The raw data for sequencing had submitted and have been deposited in the NCBI (https://submit.ncbi.nlm.nih.gov/) with accession number SUB9453991.

## Abbreviations

SCZ: Schizophrenia
16S rRNA gene sequence: 16 S ribosomal RNA gene sequence
LC-MS: Liquid chromatography-mass spectrometry
DSM-5: Diagnostic and Statistical Manual of Mental Disorders, Fifth Edition
MINI: Mini-International Neuropsychiatric Interview
PANSS: Positive and Negative Syndrome Scale
KEGG: Kyoto Encyclopedia of Genes and Genomes
HMDB: Human Metabolome Databases
BMI: Body mass index
CPZ: Chlorpromazine
PCA: Principal Component Analysis ,7-A-7-DG:7-aminomethyl-7-deazaguanine
pan_acid: Pantothenic acid
Bac_plebeius: Bacteroides_plebeius
Rum_torques: Ruminococcus_torques
Strep_sobrinus: Streptococcus_sobrinus
