# Supplementary material for: Intricate role of intestinal microbe and metabolite in schizophrenia
Source: BMC Psychiatry. 2023 Nov 17;23:856. doi: 10.1186/s12888-023-05329-z (PMC10657011; doi:10.1186/s12888-023-05329-z)
Supplement: Supplementary file 1 — Supplementary Material 1: Flow chart of the experiment and comparison of fecal metabolite content and alpha diversity of the gut microbiota between the three groups. [file 12888_2023_5329_MOESM1_ESM.docx]

Table S1

| **Fecal metabolite** | **Healthy group** | **Acute group** | **Remission group** | **H(K)** | **p** | **Acute group**  **VS**  **Remission group** | **Acute group**  **VS**  **Healthy group** | **Remission group VS**  **Healthy group** |
| --- | --- | --- | --- | --- | --- | --- | --- | --- |
| **Positive Mode** |  |  |  |  |  |  |  |  |
| 2-keto-glutaramic acid | 5.88(5.82～5.90) | 6.07(5.73～6.20) | 5.61(5.47～5.90) | 8.080 | 0.018 | 0.014 | 0.672 | 0.367 |
| L-(+)-alanine | 5.65(5.61～5.72) | 5.77(5.71～5.84) | 5.85(5.74～6.10) | 12.331 | 0.002 | 0.310 | 0.162 | 0.001 |
| L-(+)-aspartic acid | 6.36(6.17～6.63) | 5.85(5.68～6.07) | 5.98(5.82～6.02) | 14.399 | 0.001 | 1.000 | 0.001 | 0.006 |
| 5'-methylthioadenosine | 7.35(6.71～7.72) | 6.14(5.77～7.11) | 7.40(6.53～7.93) | 7.117 | 0.028 | 0.047 | 0.091 | 1.000 |
| Nicotinic acid | 9.05(8.88～9.12) | 8.64(8.28～8.88) | 9.18(8.85～9.35) | 9.285 | 0.010 | 0.015 | 0.050 | 1.000 |
| Gamma-aminobutyric acid | 6.63(6.53～6.81) | 6.68(6.54～6.86) | 7.29(7.05～7.55) | 10.646 | 0.005 | 0.017 | 0.012 | 1.000 |
| Phosphoryethanolamine | 5.73(5.70～5.79) | 6.47(5.90～6.66) | 6.02(5.83～6.44) | 10.958 | 0.004 | 1.000 | 0.008 | 0.018 |
| Ubiquinol | 6.35(6.22～6.82) | 6.35(6.20～6.62) | 6.87(6.82～6.98) | 11.061 | 0.004 | 0.060 | 0.027 | 1.000 |
| Hydroquinone | 5.83(5.79～5.87) | 6.07(5.98～6.44) | 5.97(5.86～6.21) | 8.834 | 0.012 | 0.516 | 0.009 | 0.302 |
| Testosterone | 4.79(4.74～4.88) | 5.29(4.88～5.79) | 5.12(5.03～5.21) | 10.452 | 0.005 | 1.000 | 0.008 | 0.028 |
| Deoxyadenosine | 7.17(6.68～7.48) | 6.65(6.28～6.78) | 7.31(7.00～7.86) | 10.366 | 0.006 | 0.004 | 0.137 | 0.823 |
| Pantothenic acid | 8.64(8.06～8.76) | 8.30(7.63～8.52) | 8.76(8.57～9.00) | 7.785 | 0.020 | 0.016 | 0.386 | 0.702 |
| Cinnamaldehyde | 6.23(6.03～6.56) | 5.80(5.37～6.11) | 6.10(5.75～6.34) | 7.109 | 0.029 | 0.222 | 0.028 | 1.000 |
| Dihydropteroic acid | 5.95(5.87～6.05) | 5.76(5.52～6.18) | 6.14(6.06～6.61) | 7.259 | 0.027 | 0.028 | 1.000 | 0.179 |
| Leukotriene b4 | 5.84(5.46～6.68) | 5.58(5.47～5.83) | 5.25(5.01～5.53) | 8.334 | 0.016 | 0.107 | 1.000 | 0.018 |
| Protoporphyrin ix | 6.44(6.14～7.22) | 7.38(6.99～7.95) | 6.30(6.13～7.13) | 9.071 | 0.011 | 0.015 | 0.062 | 1.000 |
| Desoxycortone | 6.35(6.10～7.00) | 7.11(6.93～7.47) | 6.92(6.43～7.04) | 7.491 | 0.024 | 0.222 | 0.022 | 1.000 |
| Sphingosylphosphorylcholine | 6.34(5.94～6.56) | 6.78(6.54～6.93) | 6.44(6.06～6.94) | 6.741 | 0.034 | 0.403 | 0.029 | 0.782 |
| 4-methyl-5-thiazoleethanol | 7.14(6.56～7.41) | 6.50(6.26～6.95) | 7.62(7.17～8.09) | 10.153 | 0.006 | 0.004 | 0.540 | 0.239 |
| Phenethylamine | 5.91(5.55～6.12) | 6.61(6.30～7.44) | 6.54(5.78～6.86) | 9.428 | 0.009 | 0.845 | 0.007 | 0.140 |
| Vitamin d2 | 6.16(5.64～6.83) | 6.01(5.54～6.47) | 5.47(5.40～5.56) | 9.604 | 0.008 | 0.038 | 1.000 | 0.014 |
| 5b-cholestane-3a,7a,12a,26-tetrol | 6.29(6.04～6.38) | 6.23(6.06～6.31) | 5.99(5.72～6.11) | 8.466 | 0.015 | 0.054 | 1.000 | 0.025 |
| 3-methoxytyramine | 5.74(5.67～5.85) | 5.78(5.70～5.94) | 5.95(5.81～6.40) | 8.898 | 0.012 | 0.122 | 1.000 | 0.012 |
| 5-methoxy-3-indoleaceate | 5.89(5.78～5.94) | 5.56(5.47～5.92) | 5.98(5.71～6.35) | 7.217 | 0.027 | 0.026 | 0.229 | 1.000 |
| Phylloquinone oxide | 5.74(5.32～5.87) | 5.20(5.13～6.44) | 5.10(5.07～5.28) | 7.131 | 0.028 | 0.129 | 1.000 | 0.036 |
| P-toluenesulfonic acid | 6.39(6.18～6.67) | 5.40(4.97～5.64) | 6.34(6.15～6.61) | 18.637 | 0.000 | 0.001 | 0.000 | 1.000 |
| 3-methylbutanoic acid | 7.16(7.00～7.25) | 6.78(6.59～6.95) | 7.08(6.95～7.16) | 12.579 | 0.002 | 0.019 | 0.003 | 1.000 |
| Paraxanthine | 6.36(5.60～6.98) | 5.34(5.07～5.99) | 5.44(5.22～5.61) | 9.993 | 0.007 | 1.000 | 0.014 | 0.021 |
| (+/-)12(13)-dihome | 8.24(7.92～8.43) | 7.99(7.41～8.30) | 8.30(8.21～8.55) | 6.094 | 0.048 | 0.041 | 0.689 | 0.689 |
| 7-aminomethyl-7-deazaguanine | 5.40(5.32～5.86) | 6.09(5.76～6.67) | 5.54(5.32～5.71) | 7.746 | 0.021 | 0.082 | 0.032 | 1.000 |
| **Negative Mode** |  |  |  |  |  |  |  |  |
| N-acetyl-d-glucosamine | 7.88(7.77～8.06) | 8.32(8.02～8.58) | 8.04(7.86～8.19) | 7.783 | 0.020 | 0.210 | 0.019 | 0.994 |
| 4-hydroxybenzoic acid | 6.37(6.28～6.57) | 6.57(6.30～6.84) | 6.27(6.18～6.39) | 6.236 | 0.044 | 0.041 | 1.000 | 0.377 |
| Citrate | 7.07(6.83～7.13) | 7.41(7.32～7.56) | 6.83(6.60～7.12) | 13.345 | 0.001 | 0.002 | 0.013 | 1.000 |
| Niacin | 7.20(7.12～7.24) | 6.98(6.70～7.08) | 7.32(6.90～7.58) | 6.557 | 0.038 | 0.047 | 0.166 | 1.000 |
| Hypoxanthine | 7.68(7.52～7.83) | 7.42(6.92～7.64) | 8.01(7.71～8.16) | 8.972 | 0.011 | 0.008 | 0.508 | 0.371 |
| Coenzyme q2 | 5.82(5.39～6.14) | 4.94(4.56～5.75) | 6.24(5.66～6.64) | 9.422 | 0.009 | 0.007 | 0.210 | 0.747 |
| Bilirubin | 6.69(6.35～6.89) | 7.05(6.89～7.26) | 6.66(6.44～6.84) | 9.459 | 0.009 | 0.019 | 0.031 | 1.000 |
| Berberine | 5.95(5.64～6.26) | 5.08(4.66～5.39) | 5.02(4.07～5.84) | 7.237 | 0.027 | 1.000 | 0.067 | 0.048 |
| Luteolin | 5.94(5.33～7.15) | 5.68(5.40～6.68) | 5.19(5.14～5.39) | 6.588 | 0.037 | 0.198 | 1.000 | 0.042 |
| N-acetylmuramic acid | 6.96(6.58～7.02) | 6.54(6.44～6.86) | 7.03(6.69～7.39) | 8.862 | 0.012 | 0.009 | 0.297 | 0.647 |
| 8z,11z,14z-eicosatrienoic acid | 6.40(6.23～6.62) | 6.95(6.77～7.38) | 6.83(6.02～7.43) | 6.939 | 0.031 | 0.845 | 0.026 | 0.345 |
| 2'-deoxyinosine | 6.93(6.41～7.56) | 6.80(5.81～7.45) | 7.69(7.37～7.79) | 9.283 | 0.010 | 0.008 | 1.000 | 0.153 |
| Hydrocinnamic acid | 6.89(6.70～7.12) | 5.90(5.59～6.18) | 7.12(6.61～7.63) | 10.942 | 0.004 | 0.009 | 0.020 | 1.000 |
| 5-hydroxyindole-3-acetic acid | 7.05(6.84～7.35) | 6.46(6.15～6.96) | 7.01(6.94～7.19) | 9.171 | 0.010 | 0.024 | 0.031 | 1.000 |
| Urobilinogen | 7.24(5.77～7.32) | 5.52(4.70～6.38) | 7.24(7.13～7.66) | 7.304 | 0.026 | 0.024 | 0.244 | 1.000 |
| Cis-5,8,11,14,17-eicosapentaenoic acid | 4.96(4.59～5.17) | 5.01(4.51～5.55) | 4.33(4.10～4.47) | 11.105 | 0.004 | 0.006 | 1.000 | 0.030 |
| Zalcitabine | 5.82(5.75～6.20) | 5.57(5.45～5.82) | 6.01(5.78～6.12) | 8.297 | 0.016 | 0.020 | 0.088 | 1.000 |
| Erucic acid | 4.66(4.54～6.21) | 5.17(4.74～5.80) | 4.33(4.14～4.62) | 8.179 | 0.017 | 0.019 | 1.000 | 0.115 |
| 1,7-dimethyluric acid | 6.86(4.76～7.36) | 4.77(4.37～5.96) | 4.47(4.33～5.04) | 6.625 | 0.036 | 1.000 | 0.295 | 0.033 |
| 1,3,7-trimethyluric acid | 4.40(3.99～5.78) | 3.93(3.65～4.09) | 3.90(3.66～4.03) | 8.069 | 0.018 | 1.000 | 0.057 | 0.028 |
| Docosapentaenoic acid | 6.76(6.17～6.99) | 7.52(7.12～7.86) | 7.02(6.33～7.33) | 10.706 | 0.005 | 0.054 | 0.005 | 1.000 |
| (13z,16z)-docosadienoic acid | 4.13(3.98～4.25) | 4.43(4.02～4.77) | 3.93(3.85～4.00) | 9.398 | 0.009 | 0.008 | 1.000 | 0.126 |

**Tab S1. comparison of Fecal metabolites content between the three groups.**


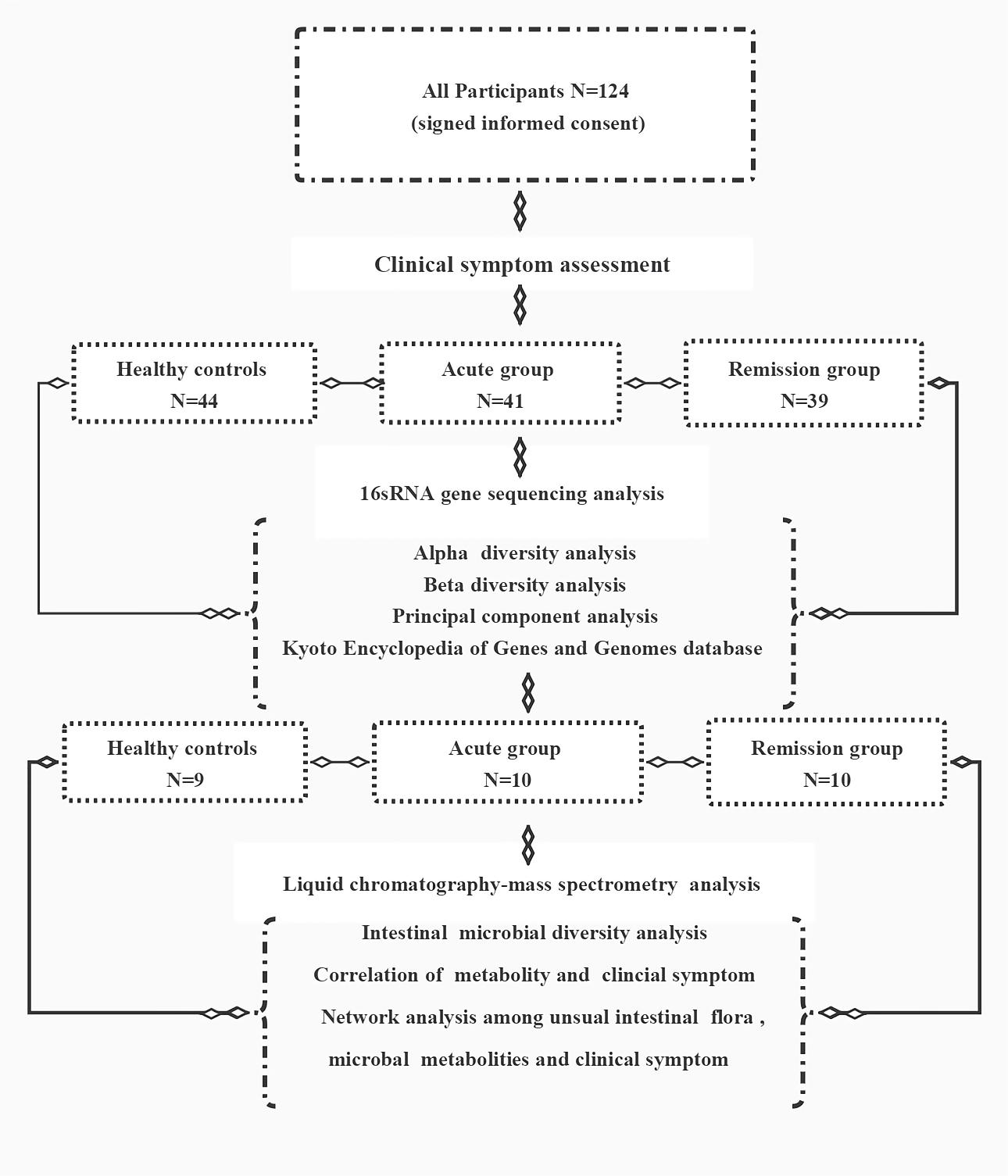


**Fig S1: Flow chart**

**
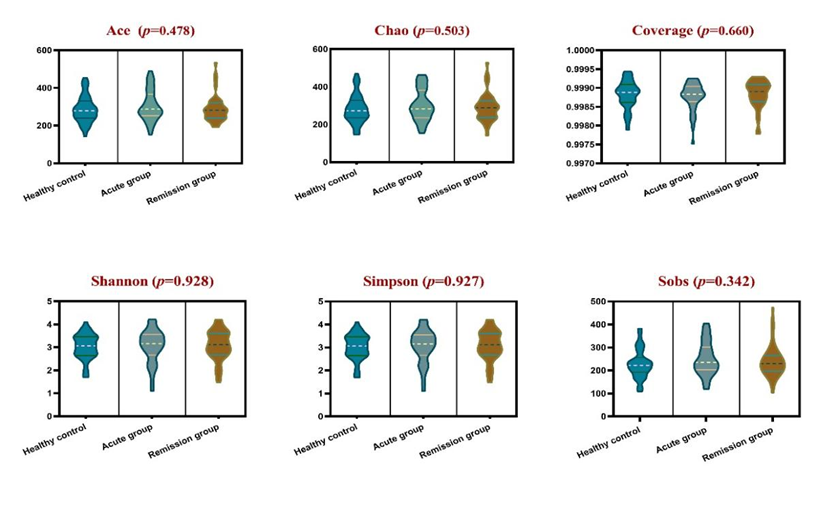
**

**Fig S2: Alpha diversity analysis**

Gut microbiome alpha-diversity analysis were estimated by the index of the Ace, Chao ,Coverage, Shannon, Simpson, and Sob indices among healthy group, acute group and remission group.
