# Supplementary material for: Intricate role of intestinal microbe and metabolite in schizophrenia
Source: BMC Psychiatry. 2023 Nov 17;23:856. doi: 10.1186/s12888-023-05329-z (PMC10657011; doi:10.1186/s12888-023-05329-z)
Supplement: Supplementary file 2 — Supplementary Material 2： Correlation between altered gut microbiota and fecal metabolites and demographic data in SCZ patients in the acute and remission groups, respectively. [file 12888_2023_5329_MOESM2_ESM.docx]

Table S2

| TabS2. Correlation between the relative abundances of altered gut microbiota and demographic data in Acute group SCZ patients. | | | | | | | | | | | | | |
| --- | --- | --- | --- | --- | --- | --- | --- | --- | --- | --- | --- | --- | --- |
| **Factors** | **Age** | | **Sex** | | **Years of education** | | **Course of disease** | | **BMI** | | **CPZ equivalent doses** | | |
|  | ***r*** | ***p*** | ***r*** | ***p*** | ***r*** | ***P*** | ***r*** | ***p*** | ***r*** | ***p*** | ***r*** | ***p*** |  |
| **Order** |  |  |  |  |  |  |  |  |  |  |  |  |  |
| Bacillales | -0.108 | 0.500 | 0.093 | 0.565 | 0.094 | 0.559 | -0.067 | 0.676 | 0.116 | 0.471 | -0.038 | 0.811 |  |
| Methylophilales | -0.023 | 0.889 | 0.133 | 0.407 | -0.092 | 0.568 | 0.051 | 0.750 | 0.086 | 0.593 | .597** | 0.000 |  |
| Methylococcales | -0.003 | 0.986 | -0.031 | 0.847 | 0.045 | 0.779 | -0.030 | 0.852 | 0.013 | 0.936 | .377* | 0.015 |  |
| **Family** |  |  |  |  |  |  |  |  |  |  |  |  |  |
| Actinomycetaceae | -0.087 | 0.590 | 0.216 | 0.175 | 0.010 | 0.952 | -0.088 | 0.583 | -0.025 | 0.878 | 0.085 | 0.598 |  |
| Staphylococcaceae | -0.108 | 0.500 | 0.093 | 0.565 | 0.094 | 0.559 | -0.067 | 0.676 | 0.116 | 0.471 | -0.038 | 0.811 |  |
| Methylophilaceae | -0.023 | 0.889 | 0.133 | 0.407 | -0.092 | 0.568 | 0.051 | 0.750 | 0.086 | 0.593 | .597** | 0.000 |  |
| Methylococcaceae | -0.003 | 0.986 | -0.031 | 0.847 | 0.045 | 0.779 | -0.030 | 0.852 | 0.013 | 0.936 | .377* | 0.015 |  |
| **Genus** |  |  |  |  |  |  |  |  |  |  |  |  |  |
| Dialister | -0.070 | 0.666 | -0.240 | 0.131 | -0.083 | 0.608 | 0.200 | 0.210 | -0.094 | 0.560 | -0.116 | 0.470 |  |
| Clostridium | 0.071 | 0.659 | 0.086 | 0.594 | -0.047 | 0.769 | -0.066 | 0.682 | -0.207 | 0.193 | -0.169 | 0.290 |  |
| Coprococcus | 0.137 | 0.392 | 0.090 | 0.577 | -.379* | 0.015 | -0.035 | 0.827 | 0.087 | 0.591 | -0.127 | 0.427 |  |
| Megasphaera | -0.131 | 0.414 | 0.139 | 0.388 | 0.040 | 0.804 | -0.120 | 0.456 | 0.020 | 0.901 | .546** | 0.000 |  |
| Staphylococcus | -0.108 | 0.500 | 0.093 | 0.565 | 0.094 | 0.559 | -0.067 | 0.676 | 0.116 | 0.471 | -0.038 | 0.811 |  |
| Aggregatibacter | 0.131 | 0.416 | 0.192 | 0.229 | -0.242 | 0.128 | .394* | 0.011 | 0.183 | 0.252 | 0.115 | 0.473 |  |
| Scardovia | 0.115 | 0.473 | 0.131 | 0.413 | -0.088 | 0.583 | 0.134 | 0.403 | -0.066 | 0.682 | -0.028 | 0.861 |  |
| Methylomonas | -0.003 | 0.986 | -0.031 | 0.847 | 0.045 | 0.779 | -0.030 | 0.852 | 0.013 | 0.936 | .377* | 0.015 |  |
| **Species** |  |  |  |  |  |  |  |  |  |  |  |  |  |
| Bacteroides_fragilis | -0.069 | 0.669 | 0.220 | 0.168 | 0.291 | 0.065 | -0.136 | 0.398 | -0.115 | 0.473 | -0.085 | 0.595 |  |
| Bacteroides_plebeius | -0.067 | 0.679 | -0.061 | 0.705 | -0.202 | 0.205 | -0.049 | 0.761 | -0.005 | 0.975 | 0.087 | 0.588 |  |
| Ruminococcus_torques | 0.183 | 0.253 | -0.139 | 0.385 | 0.197 | 0.217 | 0.010 | 0.951 | 0.002 | 0.992 | -0.080 | 0.619 |  |
| Streptococcus_sobrinus | 0.055 | 0.734 | .330* | 0.035 | -0.076 | 0.637 | 0.127 | 0.427 | .360* | 0.021 | .416** | 0.007 |  |

Note:BMI: body mass index, CPZ: chlorpromazine,**P* ≤0.05，***P*≤0.001.

In the acute group of schizophrenia, we found that seven intestinal flora which contains *Methylophilales, Methylococcales, Methylophilaceae , Methylococcaceae , Megasphaera, Methylomonas Streptococcus_sobrinus*, were positively associated with CPZ equivalent doses(*r*=0.597,*P*≤0.001;*r*=0.377,*P*=0.015; *r*=0.597,*P*≤0.001;*r*=0.377,*P*=0.015;*r*=0.546,*P*≤0.001; *r*=0.377,*P*=0.015; *r*=0.416,*P*=0.007), *Streptococcus_sobrinus* was positively correlated with sex(*r*=0.330,*P*=0.035), *Coprococcus* was negatively correlated with years of education(*r*=-0.379,*P*=0.015), *Aggregatibacter* was positively correlated with disease duration(*r*=0.394,*P*=0.011), and *Streptococcus_sobrinus* was positively correlated with BMI(*r*=0.360,*P*=0.021).

Table S3

| TabS3. Correlation between the relative abundances of altered gut microbiota and demographic data in Remission group SCZ patients. | | | | | | | | | | | | | |
| --- | --- | --- | --- | --- | --- | --- | --- | --- | --- | --- | --- | --- | --- |
| **Factors** | **Age** | | **Sex** | | **Years of education** | | **Course of disease** | | **BMI** | | **CPZ equivalent doses** | | |
|  | ***r*** | ***p*** | ***r*** | ***p*** | ***r*** | ***P*** | ***r*** | ***p*** | ***r*** | ***p*** | ***r*** | ***p*** |  |
| **Order** |  |  |  |  |  |  |  |  |  |  |  |  |  |
| Bacillales | 0.142 | 0.389 | 0.004 | 0.981 | 0.141 | 0.394 | 0.038 | 0.819 | 0.054 | 0.744 | -0.055 | 0.742 |  |
| **Family** |  |  |  |  |  |  |  |  |  |  |  |  |  |
| Actinomycetaceae | -0.079 | 0.634 | 0.252 | 0.122 | -0.072 | 0.664 | -.332* | 0.039 | 0.043 | 0.796 | 0.197 | 0.228 |  |
| Staphylococcaceae | 0.083 | 0.614 | -0.016 | 0.923 | 0.238 | 0.145 | -0.033 | 0.844 | -0.146 | 0.374 | -0.045 | 0.785 |  |
| **Genus** |  |  |  |  |  |  |  |  |  |  |  |  |  |
| Dialister | -0.010 | 0.954 | 0.085 | 0.608 | 0.176 | 0.284 | -0.022 | 0.892 | -0.233 | 0.153 | 0.110 | 0.507 |  |
| Clostridium | -0.180 | 0.273 | 0.004 | 0.979 | -0.280 | 0.085 | -.357* | 0.026 | -0.067 | 0.686 | 0.253 | 0.119 |  |
| Coprococcus | 0.013 | 0.937 | .340* | 0.034 | -0.097 | 0.558 | -0.033 | 0.843 | 0.057 | 0.732 | 0.041 | 0.806 |  |
| Megasphaera | 0.251 | 0.124 | -0.085 | 0.606 | 0.057 | 0.730 | 0.053 | 0.749 | -0.062 | 0.706 | -0.028 | 0.868 |  |
| Staphylococcus | 0.083 | 0.614 | -0.016 | 0.923 | 0.238 | 0.145 | -0.033 | 0.844 | -0.146 | 0.374 | -0.045 | 0.785 |  |
| Aggregatibacter | -0.028 | 0.864 | 0.223 | 0.172 | 0.270 | 0.096 | -0.054 | 0.745 | 0.008 | 0.960 | -0.203 | 0.215 |  |
| Scardovia | -0.082 | 0.620 | -0.080 | 0.628 | -.367* | 0.022 | -0.106 | 0.523 | 0.275 | 0.090 | -0.118 | 0.474 |  |
| Flexispira | -0.026 | 0.877 | -0.217 | 0.185 | -0.241 | 0.140 | -0.075 | 0.649 | 0.147 | 0.372 | 0.209 | 0.201 |  |
| **Species** |  |  |  |  |  |  |  |  |  |  |  |  |  |
| Bacteroides_fragilis | 0.006 | 0.972 | 0.064 | 0.698 | -0.079 | 0.634 | -0.117 | 0.478 | -0.071 | 0.669 | -0.155 | 0.347 |  |
| Bacteroides_plebeius | -.317* | 0.050 | 0.174 | 0.289 | 0.046 | 0.780 | -0.196 | 0.231 | -0.027 | 0.872 | -0.168 | 0.307 |  |
| Ruminococcus_torques | -0.131 | 0.425 | -0.057 | 0.728 | -0.206 | 0.208 | -0.194 | 0.237 | -0.047 | 0.777 | 0.025 | 0.879 |  |
| Streptococcus_sobrinus | 0.231 | 0.156 | 0.081 | 0.625 | .333* | 0.039 | -0.241 | 0.139 | -.398* | 0.012 | 0.144 | 0.383 |  |

Note:BMI: body mass index, CPZ: chlorpromazine,**P* ≤0.05，***P*≤0.001.

In the remission group of schizophrenia, *Bacteroides_plebeius* was negatively correlated with age(*r*=-0.317,*P*=0.050), Coprococcus was positively correlated with sex(*r*=0.340,*P*=0.034), Scardovia was negatively correlated with years of education(*r*=-0.367,*P*=0.022), Streptococcus_sobrinus was positively correlated with years of education(*r*=0.333,*P*=0.039), Actinomycetaceae and Clostridium were negatively correlated with disease duration(*r*=-0.332,*P*=0.039;*r*=-0.357,*P*=0.026), and Streptococcus_sobrinus was negatively correlated with BMI(*r*=-0.398,*P*=0.012).

Table S4

| TabS4. Correlation between the content of altered fecal metabolite and demographic data in Acute group SCZ patients. | | | | | | | | | | | | | |
| --- | --- | --- | --- | --- | --- | --- | --- | --- | --- | --- | --- | --- | --- |
| **Factors** | **Age** | | **Sex** | | **Years of education** | | **Course of disease** | | **BMI** | | **CPZ equivalent doses** | | |
|  | ***r*** | ***p*** | ***r*** | ***p*** | ***r*** | ***P*** | ***r*** | ***p*** | ***r*** | ***p*** | ***r*** | ***p*** |  |
| N-acetyl-d-glucosamine | .665* | 0.036 | 0.283 | 0.428 | 0.405 | 0.246 | 0.252 | 0.482 | 0.064 | 0.860 | -0.092 | 0.800 |  |
| 4-hydroxybenzoic acid | -0.193 | 0.593 | 0.073 | 0.842 | 0.350 | 0.322 | -0.467 | 0.173 | -.665* | 0.036 | -0.058 | 0.873 |  |
| Citrate | 0.024 | 0.947 | 0.406 | 0.245 | -0.442 | 0.201 | 0.183 | 0.612 | 0.006 | 0.987 | 0.339 | 0.338 |  |
| Niacin | -0.341 | 0.336 | -0.004 | 0.992 | -0.175 | 0.629 | -0.097 | 0.790 | -0.319 | 0.368 | -0.135 | 0.709 |  |
| Hypoxanthine | -0.453 | 0.188 | -0.086 | 0.813 | -0.476 | 0.164 | -0.183 | 0.614 | -0.348 | 0.325 | 0.057 | 0.877 |  |
| Coenzyme q2 | 0.038 | 0.916 | 0.086 | 0.813 | -0.249 | 0.487 | 0.299 | 0.402 | 0.315 | 0.375 | 0.511 | 0.131 |  |
| Bilirubin | -0.249 | 0.488 | -0.319 | 0.370 | -0.281 | 0.431 | 0.164 | 0.650 | 0.211 | 0.559 | -0.140 | 0.701 |  |
| Berberine | 0.087 | 0.810 | 0.214 | 0.552 | -0.318 | 0.370 | 0.403 | 0.249 | 0.422 | 0.224 | 0.582 | 0.077 |  |
| Luteolin | -0.240 | 0.505 | -0.523 | 0.121 | 0.294 | 0.410 | -0.028 | 0.938 | -0.001 | 0.997 | -0.458 | 0.183 |  |
| N-acetylmuramic acid | 0.435 | 0.209 | 0.352 | 0.318 | -0.329 | 0.353 | .780** | 0.008 | 0.583 | 0.077 | 0.227 | 0.528 |  |
| 8z,11z,14z-eicosatrienoic acid | 0.360 | 0.306 | 0.290 | 0.417 | -0.416 | 0.232 | .817** | 0.004 | .648* | 0.043 | 0.251 | 0.484 |  |
| 2'-deoxyinosine | -0.558 | 0.094 | -0.220 | 0.542 | -0.033 | 0.927 | -0.333 | 0.347 | -0.523 | 0.121 | -0.050 | 0.892 |  |
| Hydrocinnamic acid | -0.100 | 0.783 | 0.272 | 0.446 | -0.101 | 0.780 | -0.097 | 0.789 | -0.282 | 0.430 | 0.124 | 0.733 |  |
| 5-hydroxyindole-3-acetic acid | 0.127 | 0.726 | 0.260 | 0.468 | 0.402 | 0.249 | -0.414 | 0.235 | -0.465 | 0.175 | -0.029 | 0.937 |  |
| Urobilinogen | 0.351 | 0.320 | 0.292 | 0.414 | -0.432 | 0.213 | .827** | 0.003 | .660* | 0.038 | 0.261 | 0.466 |  |
| Cis-5,8,11,14,17-eicosapentaenoic acid | 0.163 | 0.653 | 0.327 | 0.356 | -0.292 | 0.414 | 0.247 | 0.491 | 0.277 | 0.438 | .724* | 0.018 |  |
| Zalcitabine | -0.191 | 0.598 | -0.018 | 0.960 | 0.307 | 0.388 | -0.398 | 0.254 | -0.518 | 0.125 | -0.168 | 0.643 |  |
| Erucic acid | 0.192 | 0.595 | 0.220 | 0.542 | 0.092 | 0.799 | 0.097 | 0.789 | -0.095 | 0.793 | 0.454 | 0.187 |  |
| 1,7-dimethyluric acid | -0.135 | 0.711 | -0.253 | 0.480 | 0.344 | 0.330 | -0.352 | 0.319 | 0.191 | 0.598 | -0.333 | 0.348 |  |
| 1,3,7-trimethyluric acid | 0.616 | 0.058 | 0.225 | 0.533 | 0.394 | 0.259 | -0.058 | 0.873 | 0.184 | 0.610 | -0.126 | 0.729 |  |
| Docosapentaenoic acid | 0.538 | 0.108 | 0.392 | 0.262 | 0.347 | 0.325 | -0.063 | 0.863 | -0.211 | 0.558 | 0.039 | 0.915 |  |
| (13z,16z)-docosadienoic acid | 0.288 | 0.421 | 0.287 | 0.421 | 0.315 | 0.375 | -0.150 | 0.679 | -0.291 | 0.415 | 0.086 | 0.814 |  |
| 2-keto-glutaramic acid | -0.217 | 0.548 | 0.095 | 0.793 | 0.027 | 0.941 | -0.248 | 0.490 | -0.438 | 0.205 | -0.064 | 0.860 |  |
| L-(+)-alanine | -0.130 | 0.720 | 0.223 | 0.535 | -0.392 | 0.262 | 0.235 | 0.514 | 0.012 | 0.973 | 0.209 | 0.563 |  |
| L-(+)-aspartic acid | -0.388 | 0.268 | -0.501 | 0.140 | 0.046 | 0.900 | 0.041 | 0.910 | -0.138 | 0.704 | -0.392 | 0.262 |  |
| 5'-methylthioadenosine | -0.531 | 0.115 | -0.565 | 0.089 | 0.408 | 0.242 | -0.503 | 0.139 | -0.476 | 0.164 | -0.481 | 0.160 |  |
| Nicotinic acid | -.632* | 0.050 | -0.358 | 0.310 | -0.090 | 0.805 | -0.355 | 0.315 | -0.483 | 0.157 | -0.285 | 0.425 |  |
| Gamma-aminobutyric acid | -0.146 | 0.687 | -0.177 | 0.625 | 0.319 | 0.369 | -0.096 | 0.792 | -0.421 | 0.226 | -0.260 | 0.467 |  |
| Phosphoryethanolamine | 0.284 | 0.427 | 0.294 | 0.409 | -0.345 | 0.328 | 0.200 | 0.579 | 0.213 | 0.554 | 0.081 | 0.824 |  |
| 2-((2e)-3,7-dimethyl-2,6-octadienyl)-5,6-dimethoxy-3-methyl-1,4-benzenediol | -0.320 | 0.368 | -0.385 | 0.272 | 0.083 | 0.819 | -0.305 | 0.391 | 0.179 | 0.621 | -0.164 | 0.651 |  |
| Hydroquinone | -0.195 | 0.588 | -0.301 | 0.399 | -0.095 | 0.793 | 0.242 | 0.500 | 0.229 | 0.525 | 0.156 | 0.667 |  |
| Testosterone | 0.456 | 0.185 | 0.359 | 0.308 | -0.445 | 0.197 | .821** | 0.004 | .680* | 0.030 | 0.270 | 0.450 |  |
| Deoxyadenosine | -0.401 | 0.251 | -0.329 | 0.354 | 0.114 | 0.753 | -0.231 | 0.521 | -0.513 | 0.130 | -0.120 | 0.740 |  |
| Pantothenic acid | -0.168 | 0.643 | 0.236 | 0.511 | -.644* | 0.044 | 0.023 | 0.949 | -0.181 | 0.618 | 0.220 | 0.541 |  |
| Cinnamaldehyde | -0.226 | 0.529 | -0.111 | 0.759 | 0.108 | 0.767 | -0.194 | 0.592 | -0.309 | 0.384 | 0.236 | 0.511 |  |
| Dihydropteroic acid | 0.502 | 0.139 | 0.246 | 0.493 | 0.614 | 0.059 | -0.348 | 0.324 | -0.457 | 0.185 | -0.183 | 0.613 |  |
| Leukotriene b4 | 0.319 | 0.370 | 0.500 | 0.141 | -0.176 | 0.626 | 0.309 | 0.385 | 0.038 | 0.917 | 0.228 | 0.527 |  |
| Protoporphyrin ix | .835** | 0.003 | 0.460 | 0.181 | 0.325 | 0.359 | 0.149 | 0.680 | 0.143 | 0.694 | -0.030 | 0.935 |  |
| Desoxycortone | 0.234 | 0.515 | 0.108 | 0.766 | -0.404 | 0.247 | .756* | 0.011 | .725* | 0.018 | 0.154 | 0.671 |  |
| (2s,3r,4e)-2-ammonio-3-hydroxy-4-octadecen-1-yl 2-(trimethylammonio)ethyl phosphate | 0.312 | 0.381 | 0.515 | 0.128 | -0.249 | 0.489 | 0.123 | 0.736 | 0.206 | 0.569 | .722* | 0.018 |  |
| 4-methyl-5-thiazoleethanol | 0.194 | 0.591 | 0.394 | 0.260 | -0.472 | 0.168 | 0.628 | 0.052 | 0.326 | 0.358 | 0.283 | 0.427 |  |
| Phenethylamine | -0.459 | 0.182 | -0.402 | 0.249 | -0.150 | 0.679 | -0.140 | 0.700 | 0.122 | 0.737 | -0.123 | 0.736 |  |
| Vitamin d2 | 0.482 | 0.158 | 0.495 | 0.145 | -0.507 | 0.135 | .885** | 0.001 | .744* | 0.014 | .636* | 0.048 |  |
| 5b-cholestane-3a,7a,12a,26-tetrol | -0.090 | 0.806 | 0.232 | 0.519 | -0.064 | 0.861 | -0.044 | 0.903 | -0.242 | 0.500 | 0.109 | 0.765 |  |
| 3-methoxytyramine | 0.176 | 0.626 | -0.023 | 0.950 | 0.593 | 0.071 | -0.386 | 0.270 | -0.538 | 0.109 | -0.318 | 0.370 |  |
| 5-methoxy-3-indoleaceate | -0.299 | 0.402 | -0.565 | 0.089 | 0.329 | 0.353 | -0.044 | 0.904 | -0.378 | 0.282 | -0.411 | 0.238 |  |
| Phylloquinone oxide | 0.406 | 0.244 | 0.407 | 0.243 | -0.086 | 0.813 | 0.180 | 0.619 | 0.266 | 0.457 | .649* | 0.042 |  |
| P-toluenesulfonic acid | -0.022 | 0.952 | 0.124 | 0.732 | 0.246 | 0.494 | -0.336 | 0.342 | -.660* | 0.038 | -0.042 | 0.908 |  |
| 3-methylbutanoic acid | -0.589 | 0.073 | -0.299 | 0.401 | -0.208 | 0.564 | -0.396 | 0.257 | -0.423 | 0.223 | -0.201 | 0.578 |  |
| Paraxanthine | 0.418 | 0.229 | 0.084 | 0.818 | 0.480 | 0.160 | -0.264 | 0.462 | 0.174 | 0.630 | -0.293 | 0.412 |  |
| (+/-)12(13)-dihome | 0.348 | 0.324 | 0.025 | 0.946 | 0.128 | 0.725 | 0.013 | 0.971 | 0.345 | 0.328 | 0.079 | 0.828 |  |
| Docosapentaenoic acid | 0.596 | 0.069 | 0.495 | 0.146 | -0.047 | 0.898 | 0.357 | 0.312 | 0.166 | 0.647 | 0.194 | 0.592 |  |
| 7-aminomethyl-7-deazaguanine | .830** | 0.003 | 0.565 | 0.089 | -0.249 | 0.488 | .723* | 0.018 | .736* | 0.015 | 0.461 | 0.180 |  |

Note:BMI: body mass index, CPZ: chlorpromazine,**p* ≤0.05，***p*≤0.001.

In the acute schizophrenia group, There are three metabolites associated with age, of which are N-acetyl-d-glucosamine, Nicotinic acid, 7-aminomethyl-7-deazaguanine(*r*=-0.665,*P*=0.036; *r*=-0.632,*P*=0.050; *r*=-0.830,*P*=0.003).Pantothenic acid negatively associated with years of education(*r*=-0.644,*P*=0.044), seven metabolites associated with duration of illness correlated, 8 metabolites correlated with BMI, and 4 metabolites correlated with chlorpromazine equivalents.

Table S5

| TabS5. Correlation between the content of altered fecal metabolite and demographic data in Remission group SCZ patients. | | | | | | | | | | | | | |
| --- | --- | --- | --- | --- | --- | --- | --- | --- | --- | --- | --- | --- | --- |
| **Factors** | **Age** | | **Sex** | | **Years of education** | | **Course of disease** | | **BMI** | | **CPZ equivalent doses** | | |
|  | ***r*** | ***p*** | ***r*** | ***p*** | ***r*** | ***P*** | ***r*** | ***p*** | ***r*** | ***p*** | ***r*** | ***p*** |  |
| N-acetyl-d-glucosamine | -0.329 | 0.353 | 0.075 | 0.836 | 0.116 | 0.750 | 0.152 | 0.675 | 0.437 | 0.207 | -0.391 | 0.264 |  |
| 4-hydroxybenzoic acid | -0.615 | 0.058 | .830** | 0.003 | 0.503 | 0.138 | -0.513 | 0.129 | 0.289 | 0.418 | 0.424 | 0.223 |  |
| Citrate | -0.216 | 0.548 | 0.073 | 0.842 | 0.484 | 0.157 | -0.106 | 0.772 | -0.059 | 0.871 | -0.024 | 0.947 |  |
| Niacin | -0.269 | 0.452 | 0.009 | 0.981 | 0.505 | 0.136 | 0.137 | 0.706 | 0.055 | 0.879 | -0.416 | 0.232 |  |
| Hypoxanthine | 0.330 | 0.351 | -0.178 | 0.623 | 0.212 | 0.556 | .696* | 0.026 | .711* | 0.021 | -.723* | 0.018 |  |
| Coenzyme q2 | 0.519 | 0.124 | -0.342 | 0.333 | -0.381 | 0.277 | 0.481 | 0.159 | 0.046 | 0.900 | -0.056 | 0.877 |  |
| Bilirubin | 0.428 | 0.217 | -0.452 | 0.190 | -0.130 | 0.720 | 0.400 | 0.252 | 0.410 | 0.239 | -0.475 | 0.166 |  |
| Berberine | -0.101 | 0.782 | 0.325 | 0.359 | -0.365 | 0.299 | -0.392 | 0.262 | -0.321 | 0.366 | .824** | 0.003 |  |
| Luteolin | -0.145 | 0.689 | -0.378 | 0.281 | -0.564 | 0.089 | -0.239 | 0.507 | -0.440 | 0.203 | 0.138 | 0.703 |  |
| N-acetylmuramic acid | -0.133 | 0.715 | 0.076 | 0.834 | 0.195 | 0.590 | 0.119 | 0.743 | 0.150 | 0.679 | -0.157 | 0.665 |  |
| 8z,11z,14z-eicosatrienoic acid | -0.078 | 0.831 | -0.244 | 0.497 | 0.102 | 0.779 | 0.095 | 0.794 | 0.264 | 0.460 | -0.359 | 0.308 |  |
| 2'-deoxyinosine | 0.009 | 0.980 | 0.112 | 0.758 | 0.207 | 0.566 | 0.016 | 0.965 | -0.034 | 0.926 | -0.107 | 0.769 |  |
| Hydrocinnamic acid | 0.329 | 0.354 | -0.163 | 0.653 | -0.149 | 0.681 | 0.413 | 0.236 | 0.118 | 0.746 | -0.189 | 0.602 |  |
| 5-hydroxyindole-3-acetic acid | -0.467 | 0.173 | 0.451 | 0.191 | 0.496 | 0.145 | -0.568 | 0.087 | -0.505 | 0.136 | 0.548 | 0.101 |  |
| Urobilinogen | -0.376 | 0.284 | -0.149 | 0.681 | -0.533 | 0.113 | -0.335 | 0.345 | -0.308 | 0.386 | 0.138 | 0.704 |  |
| Cis-5,8,11,14,17-eicosapentaenoic acid | -0.006 | 0.988 | 0.424 | 0.222 | 0.155 | 0.669 | -0.081 | 0.823 | 0.411 | 0.239 | 0.404 | 0.247 |  |
| Zalcitabine | 0.299 | 0.402 | -0.339 | 0.338 | 0.310 | 0.383 | 0.427 | 0.218 | -0.063 | 0.862 | -0.586 | 0.075 |  |
| Erucic acid | 0.472 | 0.168 | -0.336 | 0.343 | -0.319 | 0.370 | 0.490 | 0.151 | 0.124 | 0.734 | -0.155 | 0.669 |  |
| 1,7-dimethyluric acid | -0.202 | 0.575 | -0.251 | 0.484 | -0.597 | 0.069 | -0.226 | 0.530 | -0.150 | 0.680 | 0.136 | 0.707 |  |
| 1,3,7-trimethyluric acid | 0.071 | 0.846 | -0.470 | 0.171 | -0.337 | 0.340 | 0.318 | 0.371 | 0.236 | 0.511 | -0.398 | 0.254 |  |
| Docosapentaenoic acid | -0.051 | 0.889 | 0.022 | 0.953 | 0.048 | 0.895 | 0.278 | 0.437 | .699* | 0.024 | -0.391 | 0.264 |  |
| (13z,16z)-docosadienoic acid | 0.007 | 0.984 | -0.103 | 0.777 | 0.246 | 0.493 | 0.213 | 0.554 | 0.441 | 0.202 | -0.311 | 0.382 |  |
| 2-keto-glutaramic acid | -0.519 | 0.125 | 0.609 | 0.062 | 0.354 | 0.316 | -0.467 | 0.174 | -0.145 | 0.689 | 0.620 | 0.056 |  |
| L-(+)-alanine | -0.234 | 0.516 | 0.138 | 0.704 | 0.423 | 0.223 | -0.195 | 0.588 | -0.244 | 0.497 | 0.117 | 0.747 |  |
| L-(+)-aspartic acid | -0.253 | 0.480 | 0.128 | 0.724 | 0.274 | 0.444 | -0.295 | 0.408 | -0.503 | 0.138 | 0.277 | 0.438 |  |
| 5'-methylthioadenosine | -0.203 | 0.573 | 0.178 | 0.623 | 0.480 | 0.160 | 0.126 | 0.729 | 0.318 | 0.370 | -0.528 | 0.117 |  |
| Nicotinic acid | -0.307 | 0.389 | 0.320 | 0.367 | 0.422 | 0.225 | 0.095 | 0.793 | 0.318 | 0.371 | -0.324 | 0.360 |  |
| Gamma-aminobutyric acid | 0.035 | 0.925 | 0.272 | 0.447 | 0.124 | 0.733 | 0.332 | 0.348 | .882** | 0.001 | -0.248 | 0.489 |  |
| Phosphoryethanolamine | 0.242 | 0.501 | -0.488 | 0.152 | 0.297 | 0.404 | 0.415 | 0.234 | -0.072 | 0.844 | -.702* | 0.024 |  |
| 2-((2e)-3,7-dimethyl-2,6-octadienyl)-5,6-dimethoxy-3-methyl-1,4-benzenediol | -0.361 | 0.306 | 0.306 | 0.390 | 0.104 | 0.776 | -0.079 | 0.829 | 0.200 | 0.580 | -0.110 | 0.763 |  |
| Hydroquinone | 0.348 | 0.324 | -0.157 | 0.665 | 0.077 | 0.832 | 0.331 | 0.350 | -0.049 | 0.894 | -0.145 | 0.688 |  |
| Testosterone | -0.103 | 0.778 | 0.309 | 0.385 | -0.252 | 0.482 | -0.186 | 0.607 | -0.181 | 0.617 | 0.608 | 0.062 |  |
| Deoxyadenosine | 0.237 | 0.510 | 0.010 | 0.978 | -0.088 | 0.810 | 0.486 | 0.155 | .797** | 0.006 | -0.311 | 0.382 |  |
| Pantothenic acid | -0.326 | 0.358 | 0.204 | 0.572 | 0.530 | 0.115 | 0.018 | 0.961 | 0.053 | 0.884 | -0.319 | 0.369 |  |
| Cinnamaldehyde | 0.324 | 0.362 | -0.175 | 0.629 | 0.176 | 0.626 | 0.255 | 0.477 | -0.228 | 0.527 | -0.176 | 0.628 |  |
| Dihydropteroic acid | 0.195 | 0.589 | -0.084 | 0.818 | -0.040 | 0.912 | 0.421 | 0.226 | 0.520 | 0.123 | -0.317 | 0.372 |  |
| Leukotriene b4 | -0.233 | 0.518 | 0.183 | 0.612 | 0.289 | 0.417 | 0.059 | 0.870 | 0.530 | 0.115 | -0.270 | 0.450 |  |
| Protoporphyrin ix | 0.305 | 0.391 | -0.294 | 0.409 | 0.186 | 0.608 | 0.375 | 0.285 | -0.178 | 0.623 | -0.271 | 0.449 |  |
| Desoxycortone | 0.337 | 0.341 | -.702* | 0.024 | -0.147 | 0.685 | .646* | 0.043 | 0.349 | 0.323 | -.851** | 0.002 |  |
| (2s,3r,4e)-2-ammonio-3-hydroxy-4-octadecen-1-yl 2-(trimethylammonio)ethyl phosphate | 0.411 | 0.239 | 0.255 | 0.477 | 0.160 | 0.660 | 0.195 | 0.589 | 0.562 | 0.091 | -0.119 | 0.743 |  |
| 4-methyl-5-thiazoleethanol | -0.160 | 0.659 | 0.329 | 0.353 | 0.165 | 0.648 | 0.034 | 0.927 | 0.358 | 0.310 | -0.164 | 0.651 |  |
| Phenethylamine | 0.027 | 0.942 | -0.407 | 0.243 | 0.018 | 0.961 | 0.042 | 0.909 | -0.091 | 0.803 | -0.278 | 0.437 |  |
| Vitamin d2 | 0.451 | 0.191 | -0.332 | 0.349 | -0.253 | 0.481 | 0.485 | 0.155 | 0.129 | 0.722 | -0.220 | 0.542 |  |
| 5b-cholestane-3a,7a,12a,26-tetrol | -0.600 | 0.067 | 0.520 | 0.123 | .640* | 0.046 | -.713* | 0.021 | -.684* | 0.029 | 0.487 | 0.153 |  |
| 3-methoxytyramine | -0.401 | 0.250 | 0.245 | 0.496 | 0.211 | 0.558 | -0.081 | 0.825 | 0.050 | 0.890 | -0.190 | 0.598 |  |
| 5-methoxy-3-indoleaceate | -0.540 | 0.107 | 0.280 | 0.434 | 0.291 | 0.415 | -0.145 | 0.689 | 0.257 | 0.473 | -0.192 | 0.594 |  |
| Phylloquinone oxide | -0.035 | 0.923 | 0.304 | 0.394 | -0.213 | 0.554 | -0.174 | 0.630 | -0.223 | 0.535 | .680* | 0.031 |  |
| P-toluenesulfonic acid | .634* | 0.049 | -0.179 | 0.621 | 0.009 | 0.980 | 0.591 | 0.072 | 0.350 | 0.321 | -0.369 | 0.295 |  |
| 3-methylbutanoic acid | -0.081 | 0.824 | 0.351 | 0.321 | -0.149 | 0.682 | -0.252 | 0.482 | -0.161 | 0.656 | .695* | 0.026 |  |
| Paraxanthine | 0.324 | 0.361 | -0.306 | 0.390 | -.648* | 0.043 | 0.153 | 0.673 | -0.067 | 0.855 | 0.170 | 0.639 |  |
| (+/-)12(13)-dihome | -0.540 | 0.107 | 0.369 | 0.294 | -0.311 | 0.382 | -0.527 | 0.117 | -0.174 | 0.631 | .682* | 0.030 |  |
| Docosapentaenoic acid | .701* | 0.024 | -0.402 | 0.250 | -0.274 | 0.444 | .873** | 0.001 | 0.589 | 0.073 | -0.447 | 0.195 |  |
| 7-aminomethyl-7-deazaguanine | -0.079 | 0.829 | -0.348 | 0.324 | 0.084 | 0.817 | -0.051 | 0.889 | -0.162 | 0.655 | -0.231 | 0.521 |  |

Note:BMI: body mass index, CPZ: chlorpromazine,**P* ≤0.05，***P*≤0.001.

In the remission group, there are two metabolites associated with age, of which are P-toluenesulfonic acid and Docosapentaenoic acid(*r*=0.634,*P*=0.049; *r*=0.701,*P*=0.024).

There were two metabolites (4-hydroxybenzoic acid, Desoxycortone) associated with gender (*r*=0.830,*P*=0.003; *r*=-0.702,*P*=0.024),and two metabolites (5b- cholestane-3a,7a,12a,26-tetrol, Paraxanthine) associated with years of education(*r*=0.640,*P*=0.046; *r*=-0.648,*P*=0.043), 4 metabolites of which are Hypoxanthine, Desoxycortone, 5b-cholestane-3a,7a,12a,26-tetrol, Docosapentaenoic acid ,were related to the duration of the disease (*r*=0.696,*P*=0.026; *r*=0.646,*P*=0.043;*r*=-0.713,*P*=0.021; *r*=0.873,*P*=0.001).We found that there were 5 metabolites associated with BMI (Hypoxanthine, Desoxycortone, Gamma-aminobutyric acid, Deoxyadenosine, 5b-cholestane-3a,7a,12a. 26-tetrol), 6 metabolites associated with CPZ equivalent doses.
